# Controlling Lampenflora in Heritage Sites: In Situ Testing of Polyoxometalate–Ionic Liquids in the Pommery Champagne Cellar

**DOI:** 10.1002/cplu.202500043

**Published:** 2025-05-27

**Authors:** Stéphanie Eyssautier‐Chuine, Ludovic Besaury, Nathalie Vaillant‐Gaveau, Sandra Villaume, Anouck Habrant, Isabel Franco‐Castillo, Marine Rondeau, Dina Aggad, Maxime Gommeaux, Gilles Fronteau, Scott G. Mitchell

**Affiliations:** ^1^ Université de Reims Champagne‐Ardenne GEGENA 51100 Reims France; ^2^ Université de Reims Champagne‐Ardenne INRAE, FARE, UMR A 614, AFERE 51097 Reims France; ^3^ Université de Reims Champagne‐Ardenne INRAE, RIBP, USC 1488 51100 Reims France; ^4^ Instituto de Nanociencia y Materiales de Aragón (INMA‐CSIC/UNIZAR) Consejo Superior de Investigaciones Científicas‐Universidad de Zaragoza c/ Pedro Cerbuna 12 50009 Zaragoza Spain; ^5^ CIBER de Bioingeniería, Biomateriales y Nanomedicina Instituto de Salud Carlos III 28029 Madrid Spain; ^6^ Vranken‐Pommery Group 5 Place du Général Gouraud, BP1049, cedex 2 51689 Reims France; ^7^ Université de Reims Champagne‐Ardenne URCATech, MOBICYTE 51100 Reims France

**Keywords:** DNA sequencing, lampenflora, microbial communities, polyoxometalate–ionic liquids, Preventol RI80, subterranean sites

## Abstract

Artificial lighting, essential for geotouristic purposes in subterranean sites, has facilitated the growth of colored photosynthetic organisms (lampenflora) on monumental 19th century bas‐reliefs of the Pommery Champagne cellar—a UNESCO‐protected heritage site—causing significant aesthetic and physical deterioration. To sustainably preserve these stone artworks, biocidal polyoxometalate–ionic liquids (POM‐ILs) are tested alongside the commercial biocide Preventol RI80 on three trial zones: cleaned and colonized areas of a wall and clean stone samples positioned on a testing station within the cellar. After 1 year, untreated control areas exhibit growth/regrowth of biofilms, whereas surfaces treated with POM‐ILs or Preventol RI80 remain biofilm free. Measurements of colorimetry and chlorophyll fluorescence confirm the effectiveness of both biocides in controlling photosynthetic micro‐organisms. However, confocal fluorescence microscopy highlights a reduced long‐term inhibition by Preventol RI80 compared to POM‐ILs, despite the latter being applied at lower concentrations. Metagenomic analysis further validates the performance of POM‐ILs, showing a notable decrease in microbial richness and diversity in treated areas. While both products effectively inhibit phototrophs and fungi, their efficacy against Pseudomonadota is limited, likely due to microbial adaptation via antibiotic resistance genes. This study underscores the potential of POM‐ILs as a sustainable alternative for preserving cultural heritage against microbial colonization.

## Graphical abstract

The article evaluates polyoxometalate–ionic liquids (POM–ILs) as biocidal agents against lampenflora in the UNESCO‐listed Pommery Champagne cellar. POM–ILs effectively inhibit microbial colonization over one year with minimal visual impact and outperform the commercial biocide Preventol RI80. Metagenomics and microscopy confirm POM–ILs’ long‐term efficacy, suggesting their sustainable potential in cultural heritage preservation.

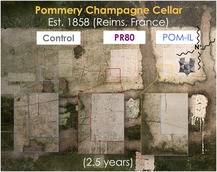

## 1 Introduction

Colonization by microbial communities is a real issue for subterranean cultural heritage due to the settlement of various micro‐organisms resulting from environmental changes brought by the touristic activity.^[^ ^1^, ^2^, ^3^ ^]^ Natural sites like caves host chemoorganotrophic micro‐organisms growing in oligotrophic but stable conditions such as darkness, low temperature, and high humidity. Such natural or pristine ecosystems are modified by the installation of artificial lights, pathways, and air circulation which promote the invasion and proliferation of external (or alien) chemolithotrophic, chemorganotrophic bacteria, fungi, and even phototrophic micro‐organisms like algae, cyanobacteria, diatoms, and moss which adapt to illuminated underground conditions, commonly referred to as lampenflora.^[^ ^1^, ^4^, ^5^, ^6^, ^7^, ^8^ ^]^

In Reims city (France), the Pommery Champagne House was reused during the 19th century, the underground previously quarried for the extraction of the chalk stone, to make a huge cellar and age Champagne. Due to this activity, this site is permanently illuminated, and walls are heavily colonized by colorful red, green, and brown biofilms around lit areas. In this context, two monumental bas‐reliefs carved in the chalk stone to celebrate Champagne and located in the tourist circuit have been illuminated by incandescent lamps for many years, increasing the local temperature. This modification was associated with stable conditions like a high relative air humidity (≈95%–99%), high‐water saturation of the stone (94.4%), and excess CO_2_ in the air released by both visitors and Champagne.^[^ ^3^, ^9^ ^]^ These environmental conditions led to the rapid colonization of lampenflora throughout the cellar.^[^ ^2^, ^10^, ^11^, ^12^ ^]^ Despite the current limitation of direct lighting on the bas‐reliefs thanks to light‐emitting diodes controlled by timers, the lampenflora which had previously settled on bas‐reliefs continues to grow. Its expansion has caused discoloration and deterioration, which has even required stone replacement, affecting the aesthetic value of this heritage as well as its long‐term sustainability.^[^ ^11^, ^13^, ^14^, ^15^, ^16^, ^17^ ^]^ Thus, active conservation of the bas‐reliefs should strike a balance between heritage preservation and local tourism interests.^[^ ^18^ ^]^ A fine‐scale study of the microbial diversity was conducted in 2020 on colorful biofilms of the lampenflora of two bas‐reliefs to characterize the microbial communities and limit their proliferation. The results showed that the communities were mainly exogenous and opportunistic, brought by the air circulation due to the activities of the wine cellar and the tourist visits.^[^ ^19^ ^]^ They were dominated by Pseudomonadota, nitrogen‐fixing *Rhizobiaceae* ^[^ ^20^ ^]^ that are responsible for stone deterioration,^[^ ^21^ ^]^ and Ascomycota like *Penicillium* sp., which were the most frequently sequenced phylum from cultured strains and are common filamentous fungi in subterranean sites. Finally, both bas‐reliefs were characterized by phototrophs like *Chromochloris sp.* algae for one and Embryophyta and *Stichococcus* sp. for the other one.

In parallel, the activity of a new generation of biocidal polyoxometalate–ionic liquids (POM–ILs), POM–IL1 and POM–IL2, was explored in laboratory assays for their efficiency against bacteria,^[^ ^22^, ^23^ ^]^ fungi,^[^ ^24^ ^]^ and algae.^[^ ^25^ ^]^ They are considered as an alternative to sodium hypochlorite and hydrogen peroxide, which are commonly used for the cleansing of cave walls, but the corrosive action and the release of chlorine gas and chloride ions are detrimental, particularly to carbonate‐confined subterranean sites.^[^ ^4^, ^26^, ^27^ ^]^ They can also replace conventional quaternary ammonium salts (QASs), which are the most widely used biocides. In a previous study, accelerated aging studies were used to evaluate the antimicrobial performance of POM–IL1 and POM–IL2 compared to the commercial Preventol RI80, which is often used in the conservation of built cultural heritage.^[^ ^28^, ^29^, ^30^, ^31^, ^32^, ^33^ ^]^

In this study, we evaluate the performance of the POM–IL1, POM–IL2, and Preventol RI80 in two illuminated locations of the Pommery cellar, where the microbial lampenflora thrives. The recurring cleanings of the bas‐reliefs by a soft airbrush with bicarbonate and a commercial biocide keep artworks clean for ≈6 months. The challenge was to assess whether POM–ILs provided a longer‐lasting efficiency than the currently used biocide. Three assays were carried out in the cellar for 1 year, two of them were preventive assays where products were applied on a cleaned chalk wall and freshly cut stone samples, and the third one demonstrated a curative approach whereby products were applied on the existing lampenflora on a wall. Monthly monitoring by in situ nondestructive techniques like chlorophyll *a* fluorescence measurement was conducted over 1 year, and stone and biofilm sampling was conducted at the end of that period for in‐depth laboratory characterization. This included microscopic observations and confocal fluorescence imaging on chalk slabs and biofilm shallow shotgun metagenome sequencing to identify and compare the overall microbial communities developed after 1 year on control surfaces and on treated surfaces with biocides. This method also enabled the prediction of the functional genes to understand how biocides impacted the metabolism of the microbes, and the Comprehensive Antibiotic Resistance Database (CARD) analysis was used to identify the antibiotic resistance genes (ARGs) developed by bacteria in response to antimicrobial treatments.

## 2 Experimental Section

### 2.1 Composition of Biocides

Three products with biocidal properties were tested in the cellar: POM‐ILs 1 and 2 and Preventol RI80 (PR). POM‐ILs 1 and 2 were validated as anticorrosive and antimicrobial coatings to safeguard stone heritage in laboratory and outdoor assays.^[^ ^25^, ^34^ ^]^ They combined molecular metal oxide anions with organic cations at room temperature. They gave the advantage that cation and anion can be tuned independently to form multifunctional mat1erials suitable for diverse surfaces. Tested POM‐ILs were based on the same lacunary Keggin polyoxoanion ([α‐SiW_11_O_39_]^8−^), whose negative charge was balanced by a tetraalkylammonium cation: tetraheptylammonium (POM‐IL1) and trihexyltetradecylammonium (POM‐IL2).^[^ ^22^ ^]^ PR (Lanxess, Köln, Germany) was a solution of QAS, alkyl‐dimethylbenzylamine chloride, in dipropylene glycol mono ether. Both POM‐ILs were dissolved in acetone to a concentration of 120 mg mL^−1^, and PR was diluted in distilled water at 5% v/v.

### 2.2 Description of the Study Sites and Assays

In March 2022, two sites were identified in the Pommery champagne cellar located in the fractured chalky underground of Reims city.^[^ ^19^, ^25^ ^]^

**Figure 1:**
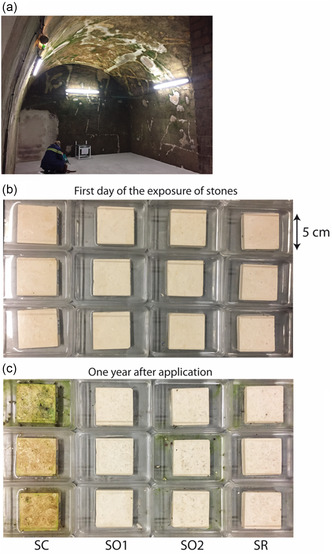
a) Photo of the illuminated room (12 h light/day) with the platform (anticolonization assay 1) located close to one of the biocolonized walls and under a neon light releasing to the stone samples with a PAR of 0.7 μmol m^−2^s^−1^. b) Triplicates of chalk stones without treatment (SC), with biocides: POM–IL1 (SO1), POM–IL2 (SO2), and Preventol RI80 (SR). c) Triplicates of stones one year after the application of products.

Assay 1 was performed on freshly cut and uncolonized stone samples, to which the products were applied in order to test their anticolonization effect. Assay 1 was thus thereafter referred to as the anticolonization assay. To this purpose, a horizontal galvanized steel platform was authorized to be settled in an empty room illuminated all day by three neon lights providing a photosynthetically active radiation (PAR) of 0.8 μmol photons m^−2^ s^−1^ at the platform level (**Figure** **1**a). Triplicate chalk samples (5 × 5 × 1 cm) were exposed on the platform comprising untreated samples as controls (named SC) and samples treated with biocides POM‐IL1 (thereafter referred to as SO1), POM‐IL2 (SO2), and PR (SR), for a total of 12 samples. Each sample was placed in a Plexiglass cup so that water could be added regularly to the bottom and the samples were wet through capillary action (Figure 1b). The room temperature and humidity were, respectively, between 11 and 15 °C and 85% all year long. In addition, the cellar walls were strongly colonized by biofilms that could foster the seeding of the samples (Figure 1a).

**Figure 2:**
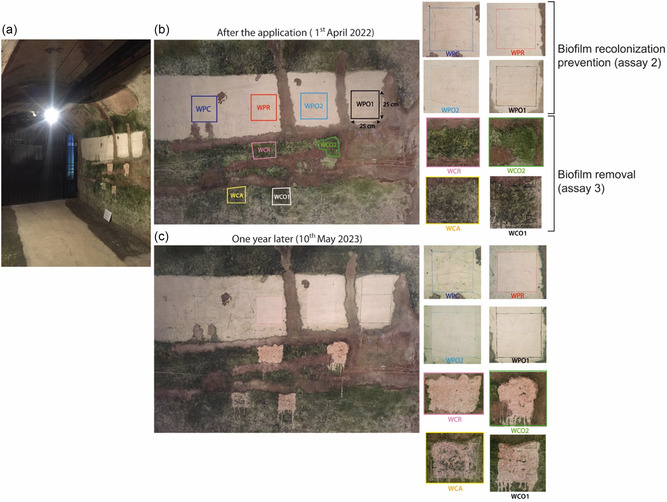
a) Photo of the illuminated corridor (12 h light/day) with biocolonized wall composed of chalk (white and green areas) and partially of cement (brown areas). A part of the chalky wall was cleaned for the biofilm recolonization prevention assay 2. b) Localization and zoom of control and treated areas on the cleaned wall for the biofilm recolonization prevention assay 2 and on the biofilm removal assay 3. c) Photo of the wall and zoom on areas of both assays 2 and 3 one year after application of products on the wall.

Assay 2 was performed on an area that was previously colonized, which was cleaned before application of the products in order to test their ability to prevent biofilm re‐establishment after cleaning. Assay 2 was thus thereafter referred to as the biofilm recolonization prevention assay. To this purpose, a biocolonized wall located in a corridor just behind the hall comprising the monumental “Fete de Bacchus” bas‐relief was investigated (**Figure** **2**a). The chalky wall was partly cemented to limit the degradation of the stone, which was naturally fractured. The assays were carried out on the chalky area. The water saturation of each tested zone, previously measured by the Karsten pipe method, was 97%–98%. For the biofilm recolonization prevention assay 2, 3 m of wall were cleaned with water and a sponge to remove the biofilm and to get a chalk clear wall on which four areas of 25 × 25 cm were defined, one was kept clear as an untreated wall (WPC), and the other areas were treated by one product each defined as WPO1 (POM‐IL1), WPO2 (POM‐IL2), and WPR (PR) (Figure 2b).

Assay 3 was performed on a biofilm‐colonized area, on which the products were applied directly, without prior removal of the biofilm, in order to test their ability to counter already‐established biofilm. Assay 3 was thus thereafter referred to as the biofilm removal assay. This assay was performed on the same wall as assay 2, on four biocolonized areas (around 25 × 15 cm) on which different products were applied and defined as WCA (acetone corresponding to a negative control), WCO1 (POM‐IL1), WCO2 (POM‐IL2), and WCR (PR). The climatic conditions were the same as in the room of assay 1 (11–15 °C and 85% relative humidity). The corridor was illuminated by a 40 W light which released a PAR of 3 μmol photons m^−2^ s^−1^ at 30 cm under the light and between 0.1 and 0.3 μmol photons m^−2^ s^−1^ on the wall.

### 2.3 Application of Biocides

For the anticolonization assay 1, the application was done on previously water‐saturated triplicate chalk samples that simulated real application conditions on a wall of the cellar. The applied quantity took into account (on the top face of each sample) the low performance of PR compared to POM–ILs obtained in the previous lab study;^[^ ^25^ ^]^ consequently, quantity was increased for the commercial product: 0.45 g of PR (corresponding to 0.18 L.m^−2^) and 0.20 g of POM‐IL solutions (corresponding to 0.10 L.m^−2^) (**Table** **1**).

**Table 1:** Concentration and applied quantity of product for the three assays.

Assay	Treatment	Concentration	Quantity[L.m^−2^]	Slab/area reference
Anticolonization assay 1	POM–IL1	120 mg mL^−1^	0.10	SO1‐1; SO1‐2; SO1‐3
	POM–IL2	120 mg mL^−1^	0.10	SO2‐1; SO2‐2; SO2‐3
	PR	5%	0.18	SR1; SR2; SR3
	CONTROL	–	–	SC1‐SC2‐SC3
Biofilm recolonization prevention assay 2	POM–IL1	120 mg mL^−1^	0.12	WPO1
	POM–IL2	120 mg mL^−1^	0.12	WPO2
	PR	5%	0.20	WPR
	CONTROL	–	–	WPC
Biofilm removal assay 3	POM–IL1	120 mg mL^−1^	0.12	WCO1
	POM–IL2	120 mg mL^−1^	0.14	WCO2
	PR	5%	0.13	WCR
	Acetone	100%	0.16	WCA

For the biofilm recolonization prevention assay 2, 8 mL of POM–IL solutions (0.12 L.m^−2^) and 10 mL of PR (0.20 L.m^−2^) were applied by brush on 25 × 25 cm cleaned and naturally wet areas.

For the biofilm removal assay 3, 5 mL of biocidal solutions were applied on naturally wet biofilm‐covered areas (between 0.12 and 0.14 L.m^−2^) with the imbibition of the biofilm by the solutions and 5 mL of only acetone on one area (0.16 L.m^−2^) to evaluate the effect of the solvent on the biofilm. The treated surfaces were not cleaned again for the duration of the assay.

### 2.4 Evaluation of Stone Color after the Application of Treatments and through the Assays

The visual aspect of the samples and wall areas tested was assessed using a Chroma Meter CR‐400 from Konica‐Minolta with a light projection tube CR‐A33c of 11 mm diameter. Calibrations were performed with a white ceramic plate CR‐A43. Three parameters determined the color location in the CIELAB color space.^[^ ^35^ ^]^ L* indicates lightness (0 = absolute black, 100 = absolute white), and a* and b* are the chromaticity coordinates. a* is the position between green (a* < 0) and red/magenta (a* > 0); b* is the position between blue (b* < 0) and yellow (b* > 0). *h*_ab_ is the hue angle (angle unit in °) which is calculated from the a* and b* parameters as *h*_ab_ = arctan(b*/a*). ΔL* and Δ*h*_ab_ were calculated and corresponded to the lightness and hue difference between the surface at the beginning of the assays and every month for each stone batch (assay 1) and each area (assay 2 and 3). Colorimetry was carried out with 10 measurements on each upper surface of samples for the anticolonization assay 1, 25 measurements on the 25 × 25 cm wall areas for the biofilm recolonization prevention assay 2, and 15 measurements on the 15 × 25 cm wall areas for the biofilm removal assay 3. The global color variation (Δ*E* ^*^_ab_) was calculated with the formulation (1) from the surfaces before application and immediately after application in the aim to assess the impact of the biocide application on the natural stone color. Then, the median and quantiles of each triplicate were calculated from 30 Δ*E* ^*^_ab_ values for assay 1, from 25 Δ*E* ^*^_ab_ values for each area of assay 2, and from 15 Δ*E* ^*^_ab_ values for each area of assay 3. The same number of measurements was also performed monthly on the three assays. Color parameters were averaged for triplicate with 30 values (assay 1) and for each area with 25 (assay 2) and 15 values (assay 3), the standard deviation and ANOVA were calculated (§ 2.10).
(1)

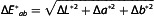




### 2.5 Microscopic Observations of Tested Surfaces after 1 Year

Observations were carried out on the samples and on the tested wall areas with dino‐lite edge digital microscope AM7915MZT with × 53 magnification. It was a nondestructive technique which does not scratch biofilms and allows direct observations of the organization of biofilms developed on the stone.

### 2.6 Photosynthetic Activity Detection

The chlorophyll *a* fluorescence of algae and plants was quantified in the cellar at the end of the assays with the MAXI‐IMAGING‐PAM Chlorophyll Fluorometer (Walz, Effeltrich, Germany). The measuring system applied a compact and powerful 300 W array of 44 Luxeon royal‐blue light‐emitting diodes (LEDs) (peak wavelength, 450 nm for the detection of fluorescence chlorophyll of eukaryotes). LED lamps provided a pulse‐modulated excitation light of 16 μmol photon^−2^ s^−1^ with a frequency of pulses adjusted to 1 Hz; the saturation pulse released 46 μmol photon m^−2^ s^−1^ with an exposure time of 720 ms. Measurements were carried out with a distance of 5 cm between the camera and the surface, corresponding to a 20 × 30 mm area and provided images of 1392 × 1040 pixels. One image was taken on each sample of the anticolonization assay 1, and four images were localized on the four corners of every area of the biofilm recolonization prevention assay 2 and the biofilm removal assay 3. The relative quantum yield of photosystem II in the photosynthetic chain (φPSII) of each image was measured and represented the number of electrons transported by a PSII reaction center per mole of quanta absorbed by PSII.

### 2.7 Confocal Laser Scanning Macroscopy and Microscopy

Morphology and autofluorescence of biofilms were observed directly on the upper surface of chalk samples (anticolonization assay 1) in the laboratory at the end of the experiment with the Confocal Axio Zoom V16 fluorescence macroscope (Zeiss, Germany) equipped with an 8‐bit RGB camera with a numerical aperture of 0.25. Fluorescence was excited and collected using led light and filters for excitation and emission: the far‐red filter set (CY5), for the detection of the photosynthetic biomass, at 640  ± 30 nm excitation, 690 ± 50 nm emission, and an exposure time of 100 ms; the blue filter set (DAPI) at 365 nm excitation, with 445 ± 50 nm emission and an exposure time of 100 ms, the green filter set (eGFP) at 470 ± 40 nm excitation, 525 nm emission, and an exposure time of 300 ms. Calcofluor White staining (1% v/v)^[^ ^36^ ^]^ was applied on the side of slabs to improve the detection of cell walls with the blue filter set at 365 nm excitation. Then, the upper surface of a control sample was scraped and observed with a fluorescence confocal microscope Leica TCS SP8 (Leica Microsystems, Germany) equipped with a 63 × oil‐immersion objective, the laser power was set to 20%, and fluorescence emission was detected using the Leica HyD hybrid detector. Three lasers were used for an excitation at 405 nm (blue), 638 nm (red), and 488 nm (green), and the acousto‐optic tunable filter was set to select emissions relative to the previous excitations: from 415 to 460 nm, from 648 to 678 nm, and from 498 to 600 nm. Images were treated using the Leica Application Suite X 3.0 (Leica Microsystems, Germany).

### 2.8 Molecular Analysis of the Microbial Diversity

#### 2.8.1 Sampling Method

Samples were scraped from every experimental surface with a scalpel cleaned with ethanol and transferred into sterile tubes and stored at −20 °C until analysis. Every slab of the anticolonization assay 1 was scraped and constituted one sample. For Assays 2 and 3, each area was divided into two parts, one half was divided into three subareas of 12.5 × 8.3 cm, which were sampled as triplicates. Three other areas of the same size were scraped from the original existing biofilm developed for many years on the wall for comparison.

#### 2.8.2 DNA Extraction

Molecular analysis of the microbial community diversity and function in biofilm samples was performed. Total genomic DNA was extracted from the different samples using PowerLyzer PowerSoil (Qiagen) following the manufacturer's protocol. The concentration and purity of the extracted DNA were assessed by spectrophotometry (Nanodrop 2000, Thermo Fisher).

#### 2.8.3 Metagenomic DNA Sequencing

The DNA were sent to Novogene (Cambridge, UK) for commercial library preparation and sequencing. Metagenomic DNA libraries were prepared using 100 ng of extracted DNA and the NEBNext Ultra DNA Library Prep Kit for Illumina (New England BioLabs), following the manufacturer's recommendations. DNA fragments were ligated to Illumina adapters and then amplified by PCR to obtain libraries ready for sequencing. The quality of the libraries was checked using a fragment analyzer (Agilent Bioanalyzer 2100). The DNA libraries were loaded onto an Illumina NovaSeq 6000 platform, generating 150 bp paired‐end reads. Sequencing was employed with a depth of ≈1 million reads per sample, allowing for the capture of overall microbial profiles.

For the sequencing analysis, raw reads were filtered to remove low‐quality sequences and adapter contamination using FastQC software.^[^ ^37^ ^]^ High‐quality reads were then aligned against a reference microbial gene microNR database^[^ ^38^ ^]^ using MetaPhlAn 3.0,^[^ ^39^ ^]^ which profiled the taxonomic composition of microbial communities in each sample. The results were normalized based on the total number of reads per sample to allow for cross‐sample comparisons. The taxonomic profiles obtained were analyzed using the R platform (version 4.1.2) and packages dedicated to metagenomic data analysis (phyloseq, vegan). Alpha diversity analyses were performed to assess microbial community richness and diversity.

Functional database of the Kyoto Encyclopedia of Genes and Genomes (KEGG)^[^ ^40^ ^]^ was used to annotate the functional genes. The presence of ARGs was identified using the CARD database. All unique genes were blasted against the CARD database (blastp, evalue ≤ 1e‐5), and the relative abundance of different resistance genes was calculated based on the results.

Despite the sampling of triplicates for each area, the sequencing could not be carried out for each of them because of too weak DNA concentration after extraction especially for samples treated with POM‐IL 1 and 2 for which enough DNA concentration was not easily reached, probably due to a weak biofilm growth. Consequently, three samples were sequenced from the original biofilm grown on the wall (WB) (Table S1, Supporting Information). For the anticolonization assay 1, sequencing was done from two control slabs (SC), two from Preventol RI80 slabs (SR), one from POM‐IL1 slab (SO1), and one from POM‐IL2 slab (SO2). For the biofilm recolonization prevention assay 2, sequencing was carried out from two control subareas (WPC), three from POM‐IL1 subareas (WPO1), one from POM‐IL2 subareas (WPO2), and three from Preventol subareas (WPR). Finally, for the biofilm removal assay 3, sequencing was done from one from POM‐IL1 subareas (WCO1), two from POM‐IL2 subareas (WCO2), no sequencing from Preventol subarea (WCR), or from acetone subarea (WCA).

#### 2.8.4 Quantitative Real‐Time PCR Assay (qPCR)

Phototrophic, fungal, and bacterial gene copy numbers were determined by quantitative PCR (qPCR) amplification of biofilm‐extracted DNA from samples collected for the shallow shotgun sequencing. Only 12 DNA extracts had enough volume to save 5 μL for qPCR (Table S1, Supporting Information). Bacterial 16S rRNA genes were amplified with the forward primer 338 F (5'‐ACTCCTACGGGAGGCAGCA‐3') and the reverse primer 806 R (5'‐GGACTACHVGGGTWTCTAAT‐3').^[^ ^41^, ^42^ ^]^ Fungal ITS1 DNA region was amplified with the forward primer ITS1‐F (5'‐TCCGTAGGTGAACCTGCGG‐3') and the reverse primer 5.8s (5'‐CGCTGCGTTCTTCATCG‐3'),^[^ ^43^, ^44^ ^]^ *rbc*L genes of the plastid genome, present in cyanobacteria and the plastids of photosynthetic eukaryotes, were used as forward primer S1F (5'‐ATGTCACCACAAACAGAGACTAAAGC‐3') and reverse primer S1R (5'‐GAAACGGTCTCTCCAACGCAT‐3').^[^ ^45^ ^]^

qPCR was performed using qPCRBIO SyGreen Blue Mix Lo‐Rox 2X (Eurobio Scientific) in 384‐well white plates (Brand) and on a CFX Opus 384 instrument (Bio‐Rad). The cycle threshold (Ct or Cq) of each amplification curve was calculated by Bio‐Rad CFX Manager Software v.4.1 using the maximum second derivative method (regression method in the software).

Optimization of qPCR parameters was performed on tenfold‐diluted pooled genomic DNA to determine the optimal primer concentration (0.1 μM) and annealing temperatures (65 °C for rbcL and ITS1, 49 °C for B16S for 15 s). Each specific fragment amplified by qPCR on the gDNA pool was extracted on electrophoresis gel and purified (NucleoSpin Gel and PCR Clean‐up, Macherey‐Nagel). They were then used to establish the standard range for B16S and *rbc*L quantification. The following equation was used to determine the number of copies of DNA template (values in italic are provided by the user). Calculations were made based on the assumption that the average molecular weight of a base pair (bp) WAS 650 Daltons. Then, the number of copies was calculated from the biofilm surface collected (cp m^2^).

Number of copies of DNA template per μL =

(2) $$=\frac{\left(\text{DNA concentration }(\text{ng}/\left(\text{μL}\right)\right)\text{ × NA}}{\left(\text{length of template }\left(\text{bp}\right)\right)\text{ × B × C}}=\frac{\left(\text{DNA concentration }(\text{ng}/(\text{μL}))\;[6.022\;\text{× }10^{23}]\right)}{\;(\text{length of template }(\text{bp})\text{ × }[1\text{ × }10^{9}]\;\text{× }650)}$$

*N*_A_ = Avogadro's number

*B* = conversion factor to ng

*C* = average weight of a base pair (Da)

Quantification was performed on 1 ng of purified gDNA. Two measurements of the gene of interest were performed for all conditions and water with a final volume of 5 μL. PCR conditions were 95 °C for 3 min, followed by 40 cycles of 5 s at 95 °C, 15 s at 65 °C (ITS1, *rbc*L), or 49 °C (B16S) and 1 min at 72 °C. For each reaction, only amplicons with the expected melting temperature, identical to the dilution range, were retained (melting program: 5 s at 60 °C to 95 °C, fluorescence capture at every 0.5 °C).

### 2.9 Statistical Analyses

Data were analyzed using the open‐source software R 4.3.1,^[^ ^46^ ^]^ with the packages R.utils,^[^ ^47^ ^]^ doBy,^[^ ^48^ ^]^ and rstatix^[^ ^49^ ^]^ for calculations, and ggplot2^[^ ^50^ ^]^ for graphics production. For colorimetric parameters, median and quantile were calculated to analyze the global color variation of the stone induced by the application of treatments. The evolution of color through time was assessed by performing Gaussian distributions of *h*_ab_ and L* data with the Shapiro–Wilk test of normality. Then, ANOVA tests were performed to define the statistical significance level *p* values between data from control stones and data from treated stones (significant difference (no star): *p* > 0.05; No significant difference *p* < 0.05 (*)) was observed. Graphics represented the averages and standard deviations of *h*_ab_ and L* parameters.

## 3 Results and Discussion

### 3.1 Global Color Change after Application of Biocides and Biological Colonization

#### 3.1.1 Immediate Effect of Product Application on Color Change

**Figure 3:**
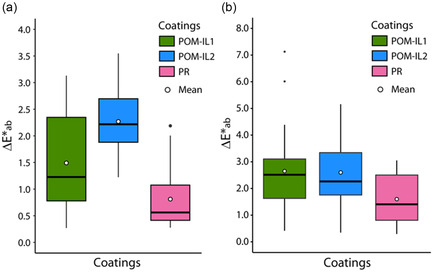
Global color change (ΔE*_ab_) of a) treatments applied on triplicate in assay 1 and b) on the wall areas in the biofilm recolonization prevention assay 2.

It is generally accepted that ΔE*_ab_ < 3 is the threshold corresponding to a visible change in surface color due to a coating product. This is especially relevant for a light‐colored stone such as chalk. In the anticolonization assay 1, ΔE*_ab_ of POM‐IL1 triplicate (SO1) displayed an asymmetric boxplot with a median at 1.2 and a mean at 1.5. 75% of values were up to 2.4, and the highest value reached 3.1 (**Figure** **3**a). ΔE*_ab_ of SO2 displayed slightly higher values than SO1, with a median and a mean very close at 2.2 and 2.3. 75% of values were below 2.7, which remained under the limit. Finally, SR ΔE*_ab_ displayed the lowest value with a median of 0.6 and a mean equal to 0.8, where 75% of the values were under 1.1. Therefore, the application of the products did not change the color enough to be visible despite a few values being higher than 3 with POM‐IL2.

In the biofilm recolonization prevention assay 2, ΔE*_ab_ was very similar for POM–ILs, with a median of 2.5 for WPO1 and 2.3 for WPO2. 75% of the values were lower than 3.1 for WPO1 and 3.3 for WPO2 (Figure 3b). Thus, the threshold was reached for both POM‐ILs, but the color change remained correct. ΔE*_ab_ median was 1.4 for PR, with 75% of the values under 2.5, which was clearly under the threshold. PR had a weaker color effect than POM‐ILs. Nevertheless, the three coatings had a weak visual impact of the color chalk, which is a white stone. In comparison, other antimicrobial coatings, including metallic nanoparticles (silver) or metal oxide nanoparticles (titanium, zinc), caused increased darkening of the building material surface with increasing metal nanoparticles concentrations (with ΔE*_ab_ threshold defined of 5, which is far higher than the conventionally accepted limit).^[^ ^51^, ^52^, ^53^, ^54^ ^]^ On the other hand, color variation associated with essential oils remained lower than 2.^[^ ^55^ ^]^ Nevertheless, the photoaging of essential oils could lead to the yellowing of the surface.^[^ ^56^, ^57^ ^]^

#### 3.1.2 Effect of Biological Colonization over Time

The analysis of the color was carried out through the most significant results obtained with the monthly variation of the lightness ΔL* and of the hue‐angle Δ*h*_ab_ between a* and b* in the chromatic sphere. During the anticolonization assay 1, there was a weak decrease of ΔL* on all treated samples, while control ΔL* decreased from week 5 with 4.9 to −22.4 at the end of the assay (Figure S1a, Supporting Information). This result testified to a darkening of the control samples (SC), whereas the treated samples remained clear. The hue variation of SC and treated samples was similar, Δ*h*_ab_ from week 1 and week 3, then values remained stable for treated samples, while it increased sharply at week 5 for controls (Figure S1b, Supporting Information). This shift of color on SC induced a greening and a browning of slabs due to the growth of the biofilm (Figure 1c). Surprisingly, the hue decreased sharply at month 7; in detail, only the hue of the third sample decreased due to the eating of the biofilm by small woodlice (Figure S2, Supporting Information).

In the biofilm recolonization prevention assay 2, WPC ΔL*WPC decreased progressively to −4.6, whereas it slightly increased on treated areas (Figure S3a, Supporting Information). WPC Δ*h*_ab_ was similar to that of treated areas until week 3; then it increased for WPC to the end of the assay, suggesting a color change by the biofilm growth on the surfaces (Figure 2c). On the contrary, the stagnation of the color parameters on treated areas suggested the absence of biofilm (Figure S3b, Supporting Information).

During the biofilm removal assay 3, ΔL* increased slightly for acetone. WCO1 ΔL* averages were higher over time than acetone, but the statistical test revealed they were similar (Figure S4a, Supporting Information), while WCO1 Δ*h*_ab_ was much more negative than WCA from the beginning of the assay, that showed a decrease of the hue due to the inhibition of the green biofilm by POM–IL1 (Figure S4b, Supporting Information Figure 2c). The result was the same for WCO2 and WCR for which ΔL* was higher than WCA and Δ*h*_ab_ much weaker, which proved a lightening and a fading due to the inhibition and the death of the biofilm on the areas treated by POM–IL2 and PR.

### 3.2 Chlorophyll *a* Fluorescence

**Figure 4:**
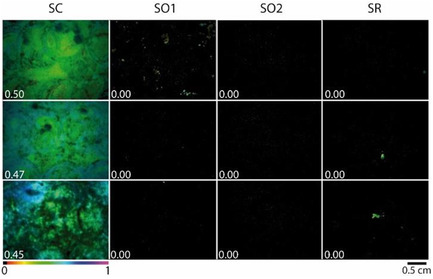
Chl. *a* fluorescence images of the upper surface of chalk stones corresponding to φPSII calculation (white numbers) in the anticolonization assay 1 after 1 year of exposure in a lighted area (12 h light/day). SC: Triplicate of untreated stones, SO1: triplicate of POM–IL1 treated stones, SO2: triplicate of POM–IL2 treated stones, SR: triplicate of Preventol RI80‐treated stones.

**Figure 5:**
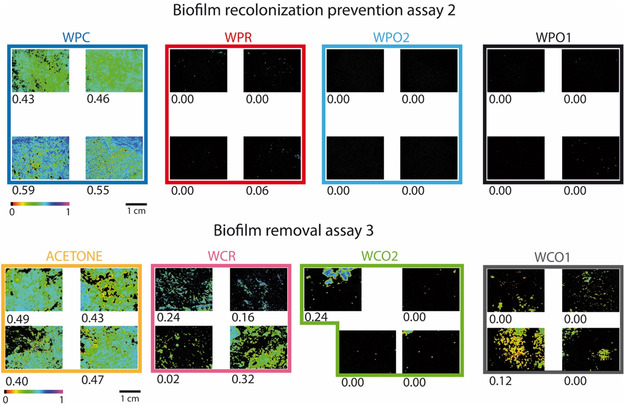
Chl. *a* fluorescence images taken at the four corners of areas of biofilm recolonization prevention assay 2 and biofilm removal assay 3 at 1 year of exposure, with φPSII calculation.

The detection of the photosynthetic activity was carried out through the chl. *a* fluorescence images and measurements at the end of the three assays. Results of both assays 1 and 2 displayed colorful images of control samples and control area. The relative quantum yield of PSII provided through φPSII was between 0.45 and 0.50 in the anticolonization assay 1 (**Figure** **4**) and between 0.43 and 0.59 in the biofilm recolonization prevention assay 2 (**Figure** **5**). Images on treated samples and areas were fully black, revealing no detection of fluorescence, thus the absence of phototrophic micro‐organisms like algae and cyanobacteria settled only on the controls. Such micro‐organisms are considered as pioneering autotrophic colonizers which contributed to the genesis of complex microbial communities by providing nutrients to heterotrophic microbes.^[^ ^58^, ^59^, ^60^ ^]^

In the biofilm removal assay 3, WCA displayed φPSII values between 0.40 and 0.49, which suggested a very weak biocidal effect of the application of acetone on the biofilm (Figure 5). On WCR, there was a decrease of φPSII, but the detection of the fluorescence (0.32) indicated that the photosynthetic micro‐organisms were alive and thriving. In WCO1 and WCO2, φPSII was barely measured, showing that their application as curative products also prevented regrowth of the biofilm even after one year.

### 3.3 Microscopic Observations in Natural Light and Confocal Laser Scanning Macro/Microscopy

In the anticolonization assay 1, SC samples were wholly green with thick brown filaments with sporophytes‐like structures associated with a green network of finer ramified filaments (Figure S5, Supporting Information). The confocal laser macroscopy highlighted the settlement of the biofilm on the stone thanks to its autofluorescence with the far‐red filter. The entire surface was covered by a dense red network of small bright red dots corresponding to the visualization of cyanobacteria and algae,^[^ ^61^, ^62^, ^63^ ^]^ while the brown network was observed with the green filter due to its nonfluorescence in the green color on the stone. Finally, this thick network was also observed with a nonfluorescence with the blue filter and smaller secondary bright fine filaments were detected. The confocal microscopy revealed autofluorescence of many coccoid morphotypes (red), which were chloroplasts inside thick filaments (blue), corresponding to cellulosic membranes. Other small tetra‐shape morphotypes were detected (green) that corresponded to cyanobacteria (Figure S6, Supporting Information).

In the biocide‐treated stones, no greening but small black dots were observed. Nevertheless, the confocal imaging revealed the covering of luminescent red dots with the far‐red filter on the entire surface of SR samples and weakly on SO1 samples, which revealed the presence of photosynthetic micro‐organisms. Nonetheless, the Preventol RI50 (5%) was shown to be more efficient than two applications of oregano essential oil (1%) since photosynthetic cells were detected under UV light when treated by this essential oil but not by the Preventol RI50.^[^ ^55^ ^]^

The tested areas on the wall were only observed in natural light microscopy. In the biofilm recolonization prevention assay 2, WPC was colonized by the same network observed in the anticolonization assay 1 with thick brown and green finer ramified filaments (Figure S7, Supporting Information). In addition, other blue‐green zones were developed, which could be cyanobacteria. On the treated areas, surfaces showed small black dots like in the anticolonization assay 1 and russet dots of iron oxidation linked to the stone composition. Moreover, on WPR, small brown filaments like the networks observed on WPC were noticed, suggesting a biological colonization of the area.

In the biofilm removal assay 3, the comparison of the treated areas with the existing biofilm displayed that the brown filament network on WCA was less dense but green and black zones still existed (Figure S8, Supporting Information). The three biocidal areas were clearer, which proved that the biocides removed many micro‐organisms including photosynthetic species. Nonetheless, the brown filamentous network remained present in all areas, and the greening was restarted only in the case of WPR, which was commensurate with the Chlorophyll fluorescence observations.

### 3.4 Quantitative PCR

From the 33 DNA extracts, only 12 were of sufficient concentration to enable qPCR analysis, in addition to the shotgun sequencing, to which priority was given. In consequence, samples with POM‐IL2 could not be analyzed at all.

The amplification with *rbc*L primers revealed that phototrophic micro‐organisms were quantified only in control samples. In the anticolonization assay 1, SC had 6.5·10^8^ cp m^2^ of bacteria genes. In the biofilm recolonization prevention assay 2, WPC had 1.5·10^8^ cp m^2^, and in the biofilm removal assay 3, WB had 9.2·10^9^ cp m^2^ of phototroph genes (Table S2, Supporting Information). Those results verified the absence of phototrophic micro‐organisms on stones with biocides. Results that are commensurate with the biocidal assays with algal and cyanobacterial strains isolated from biofilms grown in the cellar were carried out in simulated environmental conditions.^[^ ^25^ ^]^ With ITS1, the number of fungi could have been quantified only for control in the anticolonization assay 1 and in the biofilm removal assay 3, with 4.6·10^9^ cp m^2^ of fungi for SC and 3.3·10^10^ cp m^2^ for WB. Thus, biocides had a long‐lasting efficiency of 1 year against fungi. Nonetheless, in the biofilm recolonization prevention assay 2, only WPC and WPR were amplified with, respectively, 5.4·10^8^ cp m^2^ and 1.5·10^7^ cp m^2^. The quantification of fungi on the Preventol RI80‐treated area assumed a weakness of the long‐lasting efficiency of this biocide for fungi compared to both POM‐ILs.

Finally, in the anticolonization assay 1, the bacterial amount grown on the control was 2.6·10^10^ cp m^2^, while it was 1.9·10^9^ cp m^2^ on SO1 and higher on PR with 1.1·10^11^ cp m^2^. In the biofilm recolonization prevention assay 2, bacterial quantification on Preventol and POM‐IL1‐treated areas was close to the control values. The biocides had no more efficiency against bacteria after 1 year of exposure. Nonetheless, the lack of result for POM‐IL2 samples could be due to a too low DNA concentration and suggested a longer‐lasting efficiency of POM‐IL2 compared to the other biocides. The benzalkonium chloride, commonly used to eradicate micro‐organisms, has provided different efficiencies against bacteria in previous studies, ranging from the most effective effect in the inhibition of bacterial growth^[^ ^64^, ^65^ ^]^ to a weak efficiency.^[^ ^32^ ^]^

### 3.5 Molecular Analysis of the Microbial Community Diversity

The raw sequencing data have been deposited in the NCBI SRA public archive under BioProject accession number PRJNA1193470 (BioSamples SAMN45140506 to 45140527). The raw sequencing data ranged between 2.41 and 4.78 Gbp per sample. Quality control eliminated less than 0.2% of reads, indicating a good‐quality dataset. Moreover, the pan‐genome rarefaction curve reached a plateau, suggesting that the sampling strategy and sequencing depth enabled to saturate the microbial diversity in the samples (Figure S9, Supporting Information).

#### 3.5.1 Correlation Analysis of Samples

**Figure 6:**
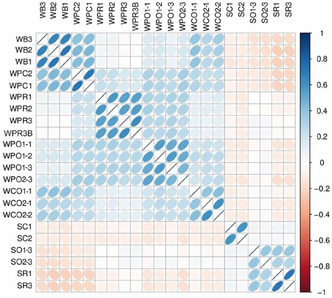
The Spearman's correlation coefficient between samples. Blue bubbles mean positive correlation. Red bubbles mean negative correlation. The darker the color, the greater the association.

The Spearman's correlation coefficients between samples were calculated. Results displayed negative or no correlation between biofilm composition of areas on the wall and on the slabs of the station (**Figure** **6**), suggesting that the microbial community composition depended primarily on the location in the cellar and/or the pristine or besmirched character of the biofilm.

High correlations (0.8) between the samples belonging to each area of the wall (assays 2 and 3) or to each batch of the anticolonization assay 1 revealed the homogeneity of the biofilm composition in every area and batch. For both preventive assays, there was a weak correlation between the 1‐year controls (SC or WPC) and the biocide‐treated areas, which indicated that the biocides modified the biological colonization of the substrate.

#### 3.5.2 Alpha Diversity Indexes

For all samples and all taxonomic levels, Good's coverage estimator values were very close to 1, and Chao1 estimator values for total diversity were very close to the observed diversity (Table 1), indicating a sufficient sequencing depth.

At the phylum level, in the anticolonization assay 1, Chao1 index values were the highest in the control slabs (SC) with 208 and 210, compared to 183 and 172 for SR (Preventol), 141 for SO1‐3 (POM‐IL1), and 129 for SO2‐3 (POM‐IL2) (**Table** **2**). Shannon index values were also the highest for SC (2.08 and 2.79) compared to SR (0.91 and 0.92) and for SO1 (0.51) and SO2 (0.25). There was a decrease in taxa distribution evenness on all treated slabs but that was more significant on SO2 than on SO1. The consequence of the POM‐ILs application was to drastically limit microbial diversity and foster the dominance of some taxa.

**Table 2:** Alpha diversity indices of samples calculated from the OTU abundance table. Chao1 is the Chao1 richness estimator, Shannon is the Shannon diversity index, and Simpson is the Simpson index.

Assay	Sample name	Observed taxa	Chao1 index	Shannon index	Good's coverage
Anticolonization assay 1	SC1	208	208	2.08	0.999
	SC2	210	210	2.79	0.999
	SO1‐3	140	141	0.51	0.999
	SO2‐3	127	129	0.25	0.999
	SR1	181	183	0.91	0.999
	SR3	172	172	0.92	0.999
Biofilm recolonization prevention assay 2	WPC1	200	200	2.26	0.999
	WPC2	202	202	2.38	0.999
	WPR1	186	186	0.58	0.999
	WPR2	188	188	0.67	0.999
	WPR3	181	182	0.58	0.999
	WPR3b	182	183	0.54	0.999
	WPO1‐1	204	205	1.51	0.999
	WPO1‐2	201	201	1.29	0.999
	WPO1‐3	192	195	0.63	0.999
	WPO2‐3	200	201	1.60	0.999
Original biofilm on wall	WB1	196	196	2.61	0.999
	WB2	202	205	2.88	0.999
	WB3	200	200	2.93	0.999
Biofilm removal assay 3	WCO1‐1	194	195	0.87	0.999
	WCO2‐1	203	204	1.70	0.999
	WCO2‐2	203	206	1.84	0.999

In the biofilm recolonization prevention assay 2, Chao1 indexes of POM‐ILs were between 195 and 205, which corresponded to those of controls, while WPR indexes were between 182 and 188. Thus, the microbial richness was similar between controls and POM‐IL areas, while it decreased for the PR area. However, Shannon indexes were weaker for every biocidal area compared to the control, which suggests a dominance of taxa.

Finally, in the biofilm removal assay 3, results were similar to the biofilm recolonization prevention assay 2. Chao1 indexes calculated on biocidal POM‐IL1 and 2 were similar to those of the existing biofilm; thus, the application of biocides after 1 year no longer affected the microbial richness. Nonetheless, the decrease of Shannon and Simpson indexes showed a dominance of taxa like in assays 1 and 2.

#### 3.5.3 Microbial Diversity Identified in the Assays

##### 3.5.3.1 Microbial Diversity on the Anticolonization Assay 1

**Figure 7:**
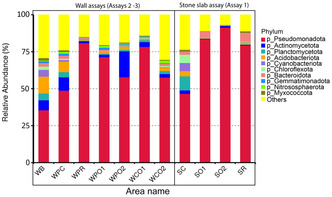
Relative taxonomy abundance of each area based on the abundance table of the plylum level, the top 10 taxa were picked out, and the other taxa were set as “Others.”

Relative taxonomy abundance displayed that bacteria were the most abundant micro‐organisms for every batch, and the abundance was higher on treated batches than on control with 0.93 on SO1, 0.95 on SO2 slabs, and 0.94 on PR and 0.87 on the control (**Figure** **7**). Pseudomonadota were the dominant phylum, especially in treated slabs with a relative abundance of 0.91 on SO2, 0.84 on SO1, and 0.79 on SR, though only 0.47 in SC. Moreover, the biodiversity was higher in SC than in treated slabs, with 0.10 for Planctomycetota, 0.06 for Chloroflexota, 0.05 for Cyanobacteriota, and 0.02 for Actinomycetota, while on treated slabs, just one phylum, Bacteroidota, was the phylum the most abundant after Pseudomonadota on SO1 and SR with, respectively, 0.04 and 0.06. Many studies have also noticed that the composition of recolonizer communities after treatment was less diverse than before treatment^[^ ^32^, ^66^ ^]^ with the replacement of specialists like Cyanobacteria, Chlorophyta, and Ascomycetes by generalists like Pseudomonadota.^[^ ^67^ ^]^

At the class level, the top 10 taxa represented an abundance of 0.60 on SC and more on treated batches with 0.91 on SO2, 0.84 on SO1, and 0.83 on SR (Figure S10, Supporting Information). Alphaproteobacteria, Betaproteobacteria, and Gammaproteobacteria were the most abundant classes in all batches. Communities in control were composed of Betaproteobacteria with *Methylibium* sp. and *Hyphomicrobium* sp., and Alphaproteobacteria with *Mesorhizobium* sp. They are common heterotrophic bacteria in outdoor soils and also underground, comprising karst soils and vermiculations due to the migration of micro‐organisms from human activities like tourists and wine workers and rainwater circulation.^[^ ^20^, ^68^, ^69^, ^70^ ^]^ Moreover, the biodiversity was higher in control than in treated slabs, and the second phyla were Planctomycetota (*Fimbriiglobus* sp.) as heterotrophic bacteria and Cyanobacteriota (*Leptolyngbia* sp.) and Chloroflexota (*Phototrophicus* sp.) as phototrophic bacteria commonly found in lampenflora.^[^ ^71^, ^72^, ^73^ ^]^

On biocide‐treated batches, Pseudomonadota genera were different according to the biocidal application: *Hyphomicrobium* sp. and *Mesorhizobium* sp. dominated on POM‐IL1 slabs, *Methylophila* sp. and *Pseudomonas* sp. on POM‐IL2 slabs, and *Phenylobacterium* sp. and *Tahibacter* sp. on PR slabs. They exclusively corresponded to Gram‐negative bacteria, a likely consequence of the quaternary ammonium previously reported,^[^ ^74^ ^]^ while on control, the biodiversity included both Gram‐negative and Gram‐positive bacteria.

**Figure 8:**
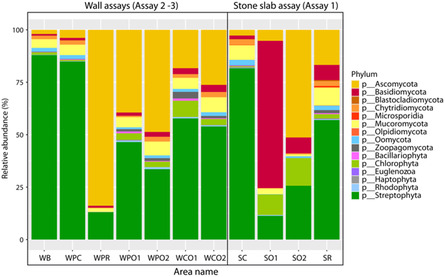
Relative taxonomy abundance of each area in phylum level for fungal and phototrophic eukaryotic microorganisms.

Despite phototrophic eukaryotes being clearly detected in imaging, the eukaryotic diversity was very poor for every batch. This result was similar to the biodiversity analyzed on biofilms, which grew on the bas‐reliefs of the cellar, bacteria wildly dominating the microbial diversity.^[^ ^19^ ^]^ Nonetheless, the relative abundance was recalculated for phototrophic eukaryotes and fungi in the aim to define which photosynthetic micro‐organisms were observed (**Figure** **8**). Here, eukaryotes on controls were mostly composed of Streptophyta with *Ceratodon* sp., which corresponded to the brown and green network observed in microscopy. SR had a higher abundance of Streptophyta than SO1 and SO2. In addition, Chlorophyta were more present on SO1 and SO2 than on SR and SC. Fungi were also sequenced secondarily on SC with Mucoromycota especially Mortierellales. On the other hand, SO1‐ and SO2‐treated batches were dominated by fungi: Basidiomycota on SO1 particularly with Polyporales and Ascomycota on SO2, with Lichinales. Finally, Ascomycota, Mucomycota, and Basidiomycota dominated on SR.

##### 3.5.3.2 Microbial Diversity on the Wall (Assays 2 and 3)

Bacteria were also the most identified micro‐organisms in both assays. The most representative phylum was Pseudomonadota as in the anticolonization assay 1. In the biofilm recolonization prevention assay 2, their abundance reached 0.49 on WPC, 0.81 on WPR, 0.71 on WPO1, and 0.58 on WPO2 (Figure 7). This phylum was more abundant on the treated areas than on the control. On WB, the existing biofilm, the biodiversity was the highest and dominated by Pseudomonadota with the weakest abundance (0.36) compared to those on treated areas. On both WB and WPC, the second phyla were developed like Acidobacteriota, Actinomycetota, Planctomycetota as heterotrophic phyla, and Cyanobacteriota (Chroococcales, Nostocales), and Chloroflexota as phototrophic bacteria. This bacterial diversity is commonly observed in karst caves,^[^ ^69^, ^70^, ^75^, ^76^, ^77^ ^]^ and this similarity with soils suggests a high adaptability of these phyla to oligotrophic conditions.^[^ ^8^, ^78^ ^]^ The main orders which dominated Pseudomonadota were Hyphomicrobiales and Rhodospirillales. Both are able to fix nitrogen to oxidize iron and manganese.^[^ ^20^, ^79^ ^]^ In vermiculations, Acidobacteria are associated with Pseudomonadota by oxidizing the reduced organic compounds provided by chemolithoautotrophic Gammaproteobacteria like Rhodospirillales.^[^ ^80^ ^]^

In areas treated with biocides, the biodiversity was almost exclusively dominated by Pseudomonadota with *Hyphomicrobium* sp. as in the control area. This result explained the decrease of Shannon index since the second phyla identified on the control did not exist anymore or was very weak. Actinomycetota with *Pseudonocardia* sp. as the second main taxa were only identified on the POM‐IL2 area, while *Mesorhizobium* sp. belonging to Pseudomonadota were the second taxa on POM‐IL1 and Preventol areas. Both are often found in caves and hypogea and are able to capture CO_2_ from the air, which is in significant quantities in the cellar with the aging of the wine and the numerous daily tourist visits.^[^ ^81^, ^82^, ^83^ ^]^ The dominance of Pseudomonadota was also found in biofilm grown on an illuminated wall treated by benzalkonium chloride or a liquorice alcoholic leaf extract. Moreover, this phyto‐derivative treatment reduced species richness, down to a single isolated strain of Pseudomonadota.^[^ ^84^ ^]^

Eukaryotes were weakly sequenced with the abundance of 0.003 on WPC, 0.002 on WPR, and 0.001 on the POM–IL‐treated areas, although a significant presence of phototrophs was strongly detected by Chlorophyll fluorescence imaging on WPC. The calculation of the relative abundance of fungi and phototrophic eukaryotes revealed a high abundance of Streptophyta with *Ceratodon sp.*, while fungi with Ascomycota dominated the biocidal areas. The Preventol RI80 area was dominated by Lichinales and Hypocreales, while Lichinales were only present as fungi on both POM‐IL areas. The Hypocreales on the Preventol area were composed of *Fusarium* sp., as has already been reported in several cave studies. This genus grows with a high ammonium content as nitrogen source^[^ ^85^ ^]^ as the consequence of the degradation of benzalkonium chloride by bacteria.^[^ ^86^ ^]^ Therefore, the occurrence of *Fusarium* sp. on the Preventol RI80 area could be associated with the composition of this treatment, which was efficient against phototrophic micro‐organisms but fostered the fungus growth.

In the biofilm removal assay 3, shallow shotgun metagenomics could only be carried out on the existing biofilm and on both POM‐IL areas, displaying a decrease of the biodiversity and the dominance of Pseudomonadota like in the other assays, with Hypocreales corresponding to *Mesorhizobium* sp. in both POM‐IL areas and *Aestuariivirga* sp. only on POM‐IL1 area. Eukaryotes were in very weak abundance, but Streptophyta were abundant and corresponded to the brown network with green fine ramification observed in the existing biofilm. This phylum was also identified in the POM‐IL area in weak abundance also observed in microscopy, as the brown network without the green ramification, probably destroyed by the biocides. Finally, fungi dominated with Ascomycota were more developed on the biocidal areas than on WB with Glomerellales. The metal oxide nanomaterials were extensively studied for their antimicrobial capacity and to inhibit the fungal growth, especially *Aspergillus niger* and *Penicillium* sp., but the efficacy depended upon the solar irradiation since they were photocatalysts^[^ ^87^, ^88^ ^]^ and upon the humidity of the substrate^[^ ^89^ ^]^ which were two major limiting factors in a subterranean site like the Pommery cellar.

#### 3.5.4 Metabolic Potential Analysis of Micro‐Organisms and Antibiotic Resistance Gene Annotation

To explore the functional potential of micro‐organisms colonizing the areas and slabs, metabolic pathways were predicted using KEGG analysis. Unique gene annotation results were used to construct a cartogram illustrating the number of annotated genes across KEGG pathway categories (Figure S11, Supporting Information). At the first hierarchical level, primary metabolism accounted for the largest number of genes, with amino acid metabolism (116,675 genes) and carbohydrate metabolism (110,073 genes) as the most prominent subcategories, followed by energy metabolism (78,065 genes) and the metabolism of cofactors and vitamins (69,858 genes). The environmental information processing category, specifically membrane transport, included 73,167 genes, while cellular processes related to prokaryotic communities comprised 66,574 genes. At the third level, 436 distinct pathways were identified. Among the top 10 most abundant functions, five were associated with the main function “Metabolism,” including glyoxylate and dicarboxylate metabolism, methane metabolism, purine metabolism, sulfur metabolism, and pyruvate metabolism. These pathways represent fundamental functions conserved across all life forms.

In the anticolonization assay 1, *Methylibium* sp. and *Methylophilus* sp. were identified as methylotrophic bacteria capable of utilizing one‐carbon (C_1_) compounds as their sole source of carbon and energy.^[^ ^90^, ^91^ ^]^ Similar to methanotrophic bacteria, which are commonly found in caves,^[^ ^78^, ^92^, ^93^ ^]^ these taxa were likely associated with methane metabolism genes (ko00680). Members of the *Rhizobiales*, such as *Hyphomicrobium* sp. and *Mesorhizobium* sp., were identified as part of carbon‐fixing bacterial communities. These taxa are associated with denitrification and the oxidation of inorganic nitrogen, with *Hyphomicrobium* species in particular being restricted to facultative methylotrophs capable of metabolizing sulfur compounds.^[^ ^21^, ^83^, ^94^, ^95^, ^96^ ^]^ At the KEGG orthology (KO) level, both control and biocide‐treated slabs exhibited functional genes predominantly associated with protein families, signaling, and cellular processes. Notably, the iron complex outer membrane receptor protein (TC.FEV.OM) was more abundant in POM‐IL‐treated slabs and even more so in slabs treated with Preventol (Figure S12, Supporting Information). Additionally, genes encoding eukaryotic‐like serine/threonine‐protein kinases were more abundant in control slabs compared to treated slabs. These kinases play a key role in signal transduction, enabling rapid responses to environmental changes.^[^ ^97^ ^]^ The observed reduction in these genes in treated slabs was attributed to the biocidal effects.

The identification of ARGs revealed that *adeF* was the main ARG detected and *qacG* was the second one, both in higher abundance on biocidal slabs than on controls (Figure S13, Supporting Information). *adeF* was an efflux pump gene encoding resistance multidrug and more specifically *qacG* for benzalkonium chloride. Therefore, genera of bacteria like *Phenylobacterium* sp. mainly present on Preventol slabs, *Methylopila* sp., and Pseudomonas on POM‐IL2 slabs acquired to be resistant to benzylkonium chloride and biocides based on quaternary ammonium compounds.^[^ ^98^, ^99^ ^]^ This resistance could explain the growth of specifically Pseudomonadota on biocidal‐treated slabs.

In the biofilm recolonization prevention assay 2, functional genes identified in controls and biocidal areas were not different, but their relative abundance was weaker in biocidal areas that were supposed to have harder environmental conditions for the development of bacteria with the biocidal treatments on the surface. Like in the anticolonization assay 1, there was a decrease of genes coding for the eukaryotic‐like serine/threonine‐protein kinase and genes implied in the production of ATP‐binding cassette (ABC) transporters related to metabolization of cyclic organic and nitrogenous compound that Pseudomonadota used to survive under oligotrophic environment like dark caves without autotrophic micro‐organisms to provide nutrients.^[^ ^100^, ^101^ ^]^ Moreover, this result was in accordance with the biocidal action of the benzalkonium chloride, which perturbed the cell wall and membrane structure that compromised the cellular permeability controls.^[^ ^29^, ^102^ ^]^ ARG analysis showed the dominance and the higher abundance on every biocidal area of *adeF* compared to control as in the anticolonization assay 1. This gene was more developed in Preventol RI80 samples than in POM‐IL1 and POM‐IL2 samples (except for 1 sample), which revealed a stronger multidrug resistance of Pseudomonadota faced with Preventol RI80 and consequently the exclusive growth of this phylum and the high quantity in this area.

In the biofilm removal assay 3, the ABC transporter as functional genes related to metabolization of cyclic organic and nitrogenous compound were significantly much more abundant on POM‐IL1 than on the POM‐IL2 area that assumed the quick adaptation of bacteria which recolonized the surface probably using remains of dead biomass and residues of biocide that provided carbon and nitrogen sources. The ARG analysis showed a similar abundance of *adeF* gene on both POM‐ILs and control, but *vanW* gene in *vanG* cluster was developed only on POM–IL1. It corresponded to a glycopeptide resistance gene cluster already linked to the presence of *Aestuariivirga* sp. This bacteria had already been identified as potential hosts for ARGs like multidrug and glycopeptide resistance in freshwater lakes.^[^ ^103^ ^]^

## 4 Conclusion

This study evaluated the performance of POM‐ILs as anticolonization biocidal coatings to mitigate lampenflora colonization in the underground Pommery Champagne cellar over a 1‐year period, comparing them with the commercial product Preventol RI80. Both biocides were effective at preventing observable color changes and chlorophyll fluorescence. However, confocal fluorescence microscopy detected phototrophic micro‐organisms and qPCR analysis quantified fungi on Preventol RI80‐treated surfaces, revealing a reduced long‐term efficacy compared to POM‐ILs. Importantly, POM‐ILs achieved these superior results with lower quantities of biocide. This result was confirmed by the observations of the wall 2.5 years after the application of the products showed the recolonization of a green biofilm only on the Preventol RI80 surface, while POM‐IL surfaces remained clean (Figure S14, Supporting Information).

Despite the effectiveness of both treatments, mild regrowth of Pseudomonadota bacteria, particularly Rhizobiales, was observed on treated stones. These bacteria, which were also dominant in control samples but alongside other bacterial phyla, did not introduce new species but rather represented a subset of the natural lampenflora. Adaptations by Rhizobiales, such as the presence of ARGs (e.g., *adeF*), likely contributed to their persistence by limiting biocide penetration. Nevertheless, this recolonization by bacteria aligns with a return to a natural microbial community on the treated stone, which cannot remain pristine indefinitely. Over time, this bacterial dominance may give way to greater biodiversity, which does not change the visual aspect of the wall after 2.5 years.

In biofilm removal applications to remove pre‐existing biofilms, Preventol RI80‐treated areas experienced rapid recolonization by phototrophs, highlighting the need for repeated applications. In contrast, POM–ILs demonstrated greater long‐term inhibitory effects, emphasizing their potential as a sustainable solution for the preservation of cultural heritage sites.

In summary, POM‐ILs demonstrate strong potential as a sustainable and efficient biocidal solution for the preservation of cultural heritage sites, outperforming commercial alternatives in long‐term inhibition of microbial growth. Future research should explore optimizing POM‐IL formulations for broader microbial targets and scaling their application to similar heritage environments, ensuring minimal ecological impact while maintaining the integrity of these historic structures.

## Conflict of Interest

The authors declare no conflict of interest.

## Supporting information

Supplementary Material

## Data Availability

The data that support the findings of this study are available in the supplementary material of this article.

## References

[1] V. Lamprinou , D. B. Danielidis , A. Pantazidou , A. Oikonomou , A. Economou‐Amilli , Int. J. Speleol. 2014, 43, 335.

[2] J. Mulec , J. Nat. Conserv. 2014, 22, 132.

[3] C. Baquedano Estévez , L. M. Merino , A. D. L. Román , J. D. Valsero , Int. J. Speleol. 2019, 48, 249.

[4] J. U. Grobbelaar , J. Appl. Phycol. 2000, 12, 309.

[5] M. Janez , K. Gorazd , J. Cave Karst Stud. 2009, 71, 109.

[6] J. Mulec , S. Kubešova , Acta Carsol. 2010, 39, 587.

[7] P. Albertano , in Ecology of Cyanobacteria II, Springer, Berlin/New York 2012, pp. 317–343.

[8] L. Alonso , T. Pommier , B. Kaufmann , A. Dubost , D. Chapulliot , J. Doré , C. J. Douady , Y. Moënne‐Loccoz , Mol. Ecol. 2019, 28, 3383. 31177607 10.1111/mec.15144

[9] G. Liger‐Belair , G. Polidori , V. Zéninari , Anal. Chim. Acta 2012, 732, 1. 22688029 10.1016/j.aca.2011.10.007

[10] J. Mulec , J. Vaupotič , J. Walochnik , Microb. Ecol. 2012, 64, 654. 22570119 10.1007/s00248-012-0059-1

[11] F. Borderie , N. Tête , D. Cailhol , L. Alaoui‐Sehmer , F. Bousta , D. Rieffel , L. Aleya , B. Alaoui‐Sossé , Sci. Total Environ. 2014, 484, 43. 24686144 10.1016/j.scitotenv.2014.03.043

[12] E. Piano , F. Bona , E. Falasco , V. La Morgia , G. Badino , M. Isaia , Sci. Total Environ. 2015, 536, 1007. 26112916 10.1016/j.scitotenv.2015.05.089

[13] F. Bastian , V. Jurado , A. Nováková , C. Alabouvette , C. Sáiz‐Jiménez , Microbiology 2010, 156, 644. 20056706 10.1099/mic.0.036160-0

[14] E. Meyer , L. D. Seale , B. Permar , A. McClary , Environ. Manage. 2017, 59, 1034. 28275851 10.1007/s00267-017-0842-3

[15] L. Bruno , L. Rugnini , V. Spizzichino , L. Caneve , A. Canini , N. T. W. Ellwood , Ann. Microbiol. 2019, 69, 1023.

[16] Z. Bontemps , L. Alonso , T. Pommier , M. Hugoni , Y. Moënne‐Loccoz , Sci. Total Environ. 2022, 816, 151492. 34793801 10.1016/j.scitotenv.2021.151492

[17] D. Isola , F. Bartoli , S. Morretta , G. Caneva , Microorganisms 2023, 11, 1770. 37512942 10.3390/microorganisms11071770 PMC10384389

[18] E. Parga‐Dans , P. A. González , R. O. Enríquez , J. Destination Marketing Manage. 2020, 18, 100499.

[19] S. Eyssautier‐Chuine , L. Besaury , N. Richet , N. Vaillant‐Gaveau , S. Laratte , M. Rondeau , C. Pierlot , A. Brunet , M. Gommeaux , Int. Biodeterior. Biodegrad. 2024, 187, 105729.

[20] R. Addesso , J. L. Gonzalez‐Pimentel , I. M. D’Angeli , J. De Waele , C. Saiz‐Jimenez , V. Jurado , A. Z. Miller , B. Cubero , G. Vigliotta , D. Baldantoni , Microb. Ecol. 2021, 81, 884. 33156395 10.1007/s00248-020-01623-5 PMC8062384

[21] M. Diaz‐Herraiz , V. Jurado , S. Cuezva , L. Laiz , P. Pallecchi , P. Tiano , S. Sanchez‐Moral , C. Saiz‐Jimenez , Sci. Rep. 2014, 4, 3610. 24402302 10.1038/srep03610 PMC3885883

[22] A. Misra , I. Franco Castillo , D. P. Müller , C. González , S. Eyssautier‐Chuine , A. Ziegler , J. M. de la Fuente , S. G. Mitchell , C. Streb , Ange. Chem., Int. Ed. 2018, 57, 14926. 10.1002/anie.201809893 30175450

[23] Q. Li , Y. Hu , B. Zhang , Carbohydr. Polym. 2021, 256, 117592. 33483078 10.1016/j.carbpol.2020.117592

[24] K. Rajkowska , A. Koziróg , A. Otlewska , M. Piotrowska , E. Atrián‐Blasco , I. Franco‐Castillo , S. G. Mitchell , Molecules 2020, 25, 5663. 33271794 10.3390/molecules25235663 PMC7729500

[25] I. Franco‐Castillo , A. Misra , S. Laratte , M. Gommeaux , R. Perarnau , N. Vaillant‐Gaveau , C. Pierlot , C. Streb , S. G. Mitchell , S. Eyssautier‐Chuine , Int. Biodeter. Biodegrad. 2022, 173, 105459.

[26] J. Iliopoulou‐Georgoudaki , A. Pantazidou , P. Theoulakis , Mémoir. Biospéol. 1993, 20, 117.

[27] J. Mulec , G. Kosi , J. Cave Karst Stud. 2009, 71, 109.

[28] S. E. Favero‐Longo , R. Benesperi , S. Bertuzzi , E. Bianchi , G. Buffa , P. Giordani , S. Loppi , P. Malaspina , E. Matteucci , L. Paoli , S. Ravera , A. Roccardi , A. Segimiro , A. Vannini , Int. Biodeterior. Biodegrad. 2017, 123, 127.

[29] A. Vannini , T. Contardo , L. Paoli , M. Scattoni , S. E. Favero‐Longo , S. Loppi , Int. Biodeterior. Biodegrad. 2018, 129, 189.

[30] M. A. Kakakhel , F. Wu , J.‐D. Gu , H. Feng , K. Shah , W. Wang , Int. Biodeterior. Biodegrad. 2019, 143, 104721.

[31] A. Marco , S. Santos , J. Caetano , M. Pintado , E. Vieira , P. R. Moreira , Build. Environ. 2020, 167, 106459.

[32] P. Sanmartín , R. Carballeira , Int. Biodeterior. Biodegrad. 2021, 156, 105130.

[33] F. Bartoli , D. Isola , A. Casanova Municchia , A. Kumbaric , G. Caneva , Front. Microbiol. 2023, 14, 1178900. 37362921 10.3389/fmicb.2023.1178900 PMC10288146

[34] S. Eyssautier‐Chuine , I. Franco‐Castillo , A. Misra , J. Hubert , N. Vaillant‐Gaveau , C. Streb , S. G. Mitchell , Sci. Total Environ. 2023, 884, 163739. 37142021 10.1016/j.scitotenv.2023.163739

[35] European committee for Standardization, CSN EN ISO 11664‐4 ‐ Colorimetry ‐ Part 4:CIE 1976 L*a*b* Colour space (ISO 11664‐ 4:2008), Category: 0117 Optics, 2011, https://www.En‐Standard.Eu.

[36] K. Herburger , A. Holzinger , Bio Prot. J. 2016, 6, 1. 10.21769/BioProtoc.1969 PMC5076763 27785458

[37] S. Andrews , FastQC: a quality control tool for high throughput sequence data. 2010, http://www.bioinformatics.babraham.ac.uk/projects/fastqc (accessed: September 2024).

[38] B. Buchfink , C. Xie , D. H. Huson , Nat. Methods 2015, 12, 59. 25402007 10.1038/nmeth.3176

[39] F. Beghini , L. J. McIver , A. Blanco‐Míguez , L. Dubois , F. Asnicar , S. Maharjan , A. Mailyan , P. Manghi , M. Scholz , A. M. Thomas , M. Valles‐Colomer , G. Weingart , Y. Zhang , M. Zolfo , C. Huttenhower , E. A. Franzosa , N. Segata , eLife 2021, 10, e65088. 33944776 10.7554/eLife.65088 PMC8096432

[40] M. Kanehisa , S. Goto , S. Kawashima , Y. Okuno , M. Hattori , Nucleic Acids Res. 2004, 32, D277. 14681412 10.1093/nar/gkh063 PMC308797

[41] Q. Liao , Y. Guo , J. Zhou , Y. Wan , R. Carballar‐Lejarazú , L. Sheng , F. Zhang , S. Wu , S. Zou , Curr. Microbiol. 2020, 77, 3321. 32939641 10.1007/s00284-020-02196-9

[42] Z. Tang , C. Huang , W. Li , W. Li , W. Tan , B. Xi , Y. Tian , L. Zhu , Chem. Eng. J. 2023, 454, 139968.

[43] M. Gardes , T. D. Bruns , Mol. Ecol. 1993, 2, 113. 8180733 10.1111/j.1365-294x.1993.tb00005.x

[44] R. Sapkota , M. Nicolaisen , Agric., Ecosyst. Environ. 2018, 257, 120.

[45] S. Radha , A. A. Fathima , S. Iyappan , M. Ramya , J. Appl. Phycol. 2013, 25, 609.

[46] R Core Team , R: A Language and Environment for Statistical Computing. R Foundation for Statistical Computing, Vienna, Austria 2023, https://www.R‐project.org/.

[47] H. Bengtsson , R.utils: Various Programming Utilities. R Package Version 2.12.3, 2023, https://CRAN.R‐project.org/package=R.utils.

[48] S. Højsgaard , S. U. Halekoh , doBy: Groupwise Statistics, LSmeans, Linear Estimates, Utilities. R Package Version 4.6.20 2023, https://CRAN.R‐project.org/package=doBy.

[49] A. Kassambara , rstatix: Pipe‐Friendly Framework for Basic Statistical Tests, R package version 0.7.2 2023, https://CRAN.R‐project.org/package=rstatix.

[50] H. Wickham , ggplot2: Elegant Graphics for Data Analysis, Springer‐Verlag New York 2016.

[51] J. Becerra , A. P. Zaderenko , M. J. Sayagués , R. Ortiz , P. Ortiz , Build. Environ. 2018, 141, 80.

[52] N. Ditaranto , S. Loperfido , I. van der Werf , A. Mangone , N. Cioffi , L. Sabbatini , Anal. Bioanal. Chem. 2011, 399, 473. 20972773 10.1007/s00216-010-4301-8

[53] T. Li , H. Zhang , X. Tan , R. Zhang , F. Wu , Q. Ma , B. Zhang , B. Su , J. Build. Eng. 2024, 98, 111043.

[54] R. Zarzuela , M. Carbú , M. L. A. Gil , J. M. Cantoral , M. J. Mosquera , Mater. Des. 2016, 114, 364.

[55] F. Antonelli , S. Iafrate , M. Tescari , M. Giandomenico , A. Kumbaric , M. Bartolini , Microorganisms 2024, 12, 1520. 39203363 10.3390/microorganisms12081520 PMC11356633

[56] M. Veneranda , L. Blanco‐Zubiaguirre , G. Roselli , G. Di Girolami , K. Castro , J. M. Madariaga , Microchem. J. 2018, 138, 1.

[57] H. Yıldız Acar , H. Sert 2022, Use and Effect of Essential Oils in The Maintenance and Preservation of Historical Monuments, SRRN, 10.2139/ssrn.4248889.

[58] S. Popović , G. Subakov Simić , M. Stupar , N. Unković , D. Predojević , J. Jovanović , M. Ljaljević Grbić , Int. J. Speleol. 2015, 44, 4.

[59] P. Di Martino , Open Conf. Proc. J. 2016, 7, 52.

[60] M. Romani , E. Adouane , C. Carrion , C. Veckerlé , D. Boeuf , F. Fernandez , M. Lefèvre , L. Intertaglia , A. M. S. Rodrigues , P. Lebaron , R. Lami , Int. Biodeterior. Biodegrad. 2021, 162, 105230.

[61] M. Roldán , C. Ascaso , J. Wierzchos , Appl. Environ. Microbiol. 2014, 80, 2998. 24610843 10.1128/AEM.03428-13 PMC4018928

[62] C. C. Gaylarde , P. M. Gaylarde , B. A. Neilan , Curr. Microbiol. 2012, 65, 183. 22614098 10.1007/s00284-012-0123-6

[63] J. R. Lawrence , T. R. Neu , G. D. W. Swerhone , J. Microbiol. Methods 1998, 32, 253.

[64] T. Li , Y. Hu , B. Zhang , J. Cult. Heritage 2020, 43, 45.

[65] D. Pinna , J. Cult. Heritage 2023, 61, 217.

[66] C. Urzì , L. De , L. Krakova , D. Pangallo , L. Bruno , Sci. Total Environ. 2016, 572, 252. 27501424 10.1016/j.scitotenv.2016.07.195

[67] C. Zhu , L. Wang , B. Wang , B. Wang , M. Tang , X. Wang , Q. Li , Y. Hu , B. Zhang , Int. Biodeterior. Biodegrad. 2023, 178, 105569.

[68] K. Tomczyk‐Żak , U. Zielenkiewicz , Geomicrobiol. J. 2016, 33, 20.

[69] C. Oliveira , L. Gunderman , C. A. Coles , J. Lochmann , M. Parks , E. Ballard , G. Glazko , Y. Rahmatallah , A. J. Tackett , D. J. Thomas , Diversity 2017, 9, 31. 29551950 10.3390/d9030031 PMC5856467

[70] J. Ai , J. Guo , Y. Li , X. Zhong , Y. Lv , J. Li , A. Yang , Environ. Sci. Pollut. Res. 2022, 29, 25858. 10.1007/s11356-021-17783-x 34854002

[71] A. Popkova , S. Mazina , T. Lashenova , Ecol. Monten. 2019, 23, 8.

[72] N. Nikolić , N. Zarubica , B. Gavrilović , D. Predojević , I. Trbojević , G. Subakov‐Simić , S. Popović , J. Cave Karst Stud. 2020, 82, 69.

[73] S. E. Mazina , T. V. Gasanova , E. V. Kozlova , A. V. Popkova , A. S. Fedorov , I. L. Bukharina , A. S. Pashkova , M. V. Larionov , Life 2023, 13, 164. 36676113 10.3390/life13010164 PMC9863006

[74] E. V. Akatova , J. M. González Grau , C. Sáiz‐Jiménez , Coalition 2007, 14, 2.

[75] V. Jurado , J. L. Gonzalez‐Pimentel , A. Z. Miller , B. Hermosin , I. M. D’Angeli , P. Tognini , J. De Waele , C. Saiz‐Jimenez , Front. Earth Sci. 2020, 8, 586248.

[76] H. Kelly , M. N. Spilde , D. S. Jones , P. J. Boston , Life 2021, 11, 59. 33467599 10.3390/life11010059 PMC7830032

[77] A. G. Bendia , F. Callefo , M. N. Araújo , E. Sanchez , V. C. Teixeira , A. Vasconcelos , G. Battilani , V. H. Pellizari , F. Rodrigues , D. Galante , Astrobiology 2022, 22, 293. 34694925 10.1089/ast.2021.0016

[78] K. H. Lavoie , A. S. Winter , K. J. H. Read , E. M. Hughes , M. N. Spilde , D. E. Northup , PLOS ONE 2017, 12, e0169339. 28199330 10.1371/journal.pone.0169339 PMC5310854

[79] N. Tsouggou , A. Oikonomou , K. Papadimitriou , P. N. Skandamis , Microorganisms 2023, 11, 2681. 38004693 10.3390/microorganisms11112681 PMC10673238

[80] D. B. Meisinger , J. Zimmermann , W. Ludwig , K.‐H. Schleifer , G. Wanner , M. Schmid , P. C. Bennett , A. S. Engel , N. M. Lee , Environ. Microbiol. 2007, 9, 1523. 17504489 10.1111/j.1462-2920.2007.01271.x

[81] M. Diaz‐Herraiz , V. Jurado , S. Cuezva , L. Laiz , P. Pallecchi , P. Tiano , S. Sanchez‐Moral , C. Saiz‐Jimenez , Sci. Rep. 2013, 3, 1440. 23486535 10.1038/srep01440 PMC3595702

[82] L. Krakova , F. De Leo , L. Bruno , D. Pangallo , C. Urzì , Environ. Microbiol. 2015, 17, 1738. 25244154 10.1111/1462-2920.12626

[83] F. Antonelli , S. Lafrate , M. Tescari , M. Giandomenico , A. Kumbaric , M. Bartolini , Microorganisms 2024, 12, 1520. 39203363 10.3390/microorganisms12081520 PMC11356633

[84] L. Rugnini , G. Migliore , F. Tasso , N. T. W. Ellwood , A. R. Sprocati , L. Bruno , Appl. Sci. 2020, 10, 6584.

[85] F. Stomeo , M. C. Portillo , J. M. Gonzalez , Curr. Microbiol. 2009, 59, 321. 19536596 10.1007/s00284-009-9437-4

[86] F. Bastian , C. Alabouvette , V. Jurado , C. Saiz‐Jimenez , Naturwissenschaften 2009, 96, 863. 19404600 10.1007/s00114-009-0540-y

[87] M. Ben Chobba , M. L. Weththimuni , M. Messaoud , C. Urzi , M. Licchelli , Coatings 2024, 14, 203. 10.1007/s11356-019-04680-7 30864042

[88] D. Pinna , Int. Biodeterior. Biodegrad. 2022, 172, 105437.

[89] S. A. Ruffolo , F. De Leo , M. Ricca , A. Arcudi , C. Silvestri , L. Bruno , C. Urzì , M. F. La Russa , Int. Biodeterior. Biodegrad. 2017, 123, 17.

[90] D. Wischer , D. Kumaresan , A. Johnston , M. El Khawand , J. Stephenson , A. M. Hillebrand‐Voiculescu , Y. Chen , J. Colin Murrell , ISME J. 2015, 9, 195. 25050523 10.1038/ismej.2014.102 PMC4274414

[91] Z. E. Havlena , L. D. Hose , H. R. DuChene , G. M. Baker , J. D. Powell , A. L. Labrado , B. Brunner , D. S. Jones , Geobiology 2024, 22, e12594. 38700397 10.1111/gbi.12594

[92] D. Kumaresan , D. Wischer , J. Stephenson , A. Hillebrand‐Voiculescu , J. C. Murrell , Geomicrobiol. J. 2014, 31, 186.

[93] T. Martin‐Pozas , J. L. Gonzalez‐Pimentel , V. Jurado , S. Cuezva , I. Dominguez‐Moñino , A. Fernandez‐Cortes , J. C. Cañaveras , S. Sanchez‐Moral , C. Saiz‐Jimenez , Appl. Sci. 2020, 10, 8130.

[94] F. Liao , Sci. Total Environ. 2020, 748, 141317. 32814290 10.1016/j.scitotenv.2020.141317

[95] X. Wang , W. Li , Y. Xiao , A. Cheng , T. Shen , M. Zhu , L. Yu , CATENA 2021, 204, 105418.

[96] A. Z. Miller , A. M. García‐Sánchez , M. L. Coutinho , M. F. Costa Pereira , F. Gázquez , J. M. Calaforra , P. Forti , J. Martínez‐Frías , T. Toulkeridis , A. T. Caldeira , C. Saiz‐Jimenez , Coatings 2020, 10, 1134.

[97] C. Zhang , W. Sun , M. Tan , M. Dong , W. Liu , T. Gao , L. Li , Z. Xu , R. Zhou , Front. Cell. Infect. Microbiol. 2017, 7, 66. 28326294 10.3389/fcimb.2017.00066 PMC5339665

[98] J. Luo , Y. Xu , J. Wang , L. Zhang , X. Jiang , J. Shen , J. Environ. Sci. 2021, 108, 134. 10.1016/j.jes.2021.02.017 34465427

[99] Q. Li , C. Wu , J. He , B. Zhang , Int. Biodeterior. Biodegrad. 2023, 185, 105688.

[100] M. Ortiz , A. Legatzki , J. W. Neilson , B. Fryslie , W. M. Nelson , R. A. Wing , C. A. Soderlund , B. M. Pryor , R. M. Maier , ISME J. 2014, 8, 478. 24030597 10.1038/ismej.2013.159 PMC3906820

[101] S. De Mandal , R. Chatterjee , N. S. Kumar , BMC Microbiol. 2017, 17, 90. 28399822 10.1186/s12866-017-1002-x PMC5387202

[102] M. Mitova , M. Iliev , A. Nováková , A. Gorbushina , V. Groudeva , P. Martin‐Sanchez , Int. J. Speleol. 2017, 46, 67.

[103] C. Mao , X. Wang , X. Li , Q. Kong , E. G. Xu , J. Huang , J. Hazard. Mater. Adv. 2023, 9, 100233.

