# Supplementary material for: Controlling Lampenflora in Heritage Sites: In Situ Testing of Polyoxometalate–Ionic Liquids in the Pommery Champagne Cellar
Source: Chempluschem. 2025 May 27;90(7):e202500043. doi: 10.1002/cplu.202500043 (PMC12261054; doi:10.1002/cplu.202500043)
Supplement: Supplementary file 1 — Supplementary Material [file CPLU-90-e202500043-s001.pdf]

Supplementary information

Controlling Lampenflora in Heritage Sites: In Situ Testing of POM-ILs in the Pommery Champagne Cellar

Stéphanie Eyssautier-Chuine[a]*, Ludovic Besaury[b], Nathalie Vaillant-Gaveau[c], Sandra Villaume[c], Anouk Habrant[b], Isabel Franco-Castillo[d][e], Marine Rondeau[f], Dina Aggad[g] Maxime Gommeaux[a], Gilles Fronteau[a], Scott G. Mitchell [d][e]

[a] Université de Reims Champagne-Ardenne, GEGENA, 51100, Reims, France. * Email: stephanie.eyssautier@univ-reims.fr
[b] Université de Reims Champagne Ardenne, INRAE, FARE, UMR A 614, Chaire AFERE, 51097, Reims, France.
[c] Université de Reims Champagne-Ardenne, INRAE, RIBP, USC 1488, 51100, Reims, France.
[d] Instituto de Nanociencia y Materiales de Aragón (INMA-CSIC/UNIZAR), Consejo Superior de Investigaciones Científicas-Universidad de Zaragoza, c/ Pedro Cerbuna 12, 50009, Zaragoza, Spain. Email : scott.mitchell@csic.es
[e] CIBER de Bioingeniería, Biomateriales y Nanomedicina, Instituto de Salud Carlos III, 28029, Madrid, Spain.
[f] Vranken-Pommery Group, 5 Place du Général Gouraud, BP1049, 51689, Reims, cedex 2, France
[g] Université de Reims Champagne Ardenne, URCATech - MOBICYTE, 51100, Reims, France.

Table S1. Description of areas and samples of the three assays: **X** corresponds to samples which were sequenced in Shotgun sequencing and analyzed in qPCR. 0 corresponds to the absence of sequencing and qPCR because of a too weak DNA concentration and volume.

Assay area	Area name	Sample name	Shotgun sequencing	qPCR
**Anti-colonization assay 1**				
Control slabs without coating	SC	SC1	**X**	**X**
		SC2	**X**	**X**
		SC3	0	0
Slabs treated with Preventol RI80®	SR	SR1	**X**	**X**
		SR2	0	0
		SR3	**X**	**X**
Slabs treated with POM-IL1	SO1	SO1-1	0	0
		SO1-2	0	0
		SO1-3	**X**	**X**
Slabs treated with POM-IL2	SO2	SO2-1	0	0
		SO2-2	0	0
		SO2-3	**X**	0
**Biofilm recolonization prevention assay 2**				
Original biofilm	WB	WB1	**X**	0
		WB2	**X**	**X**
		WB3	**X**	**X**
Cleaned control area without coating	WPC	WPC1	**X**	0
		WPC2	**X**	**X**
		WPC3	0	0
Cleaned area treated with Preventol RI80®	WPR	WPR1	**X**	**X**
		WPR2	**X**	0
		WPR3	**X**	**X**
		WPR3b	**X**	0
Cleaned area treated with POM-IL1	WPO1	WPO1-1	**X**	0
		WPO1-2	**X**	**X**
		WPO1-3	**X**	0
Cleaned area treated with POM-IL2	WPO2	WPO2-1	0	0
		WPO2-2	0	0
		WPO2-3	**X**	0
**Biofilm removal assay 3**				
Biocolonized wall treated with acetone	WCA	WCA1	0	0
		WCA2	0	0
		WCA3	0	0

Biocolonized wall treated with Preventol RI80	WCR	WCR1	0	0
		WCR2	0	0
		WCR3	0	0
Biocolonized wall treated with POM-IL1	WCO1	WCO1-1	**X**	**X**
		WCO1-2	0	0
		WCO1-3	0	0
Biocolonized wall treated with POM-IL2	WCO2	WCO2-1	**X**	0
		WCO2-2	**X**	0
		WCO2-3	0	0

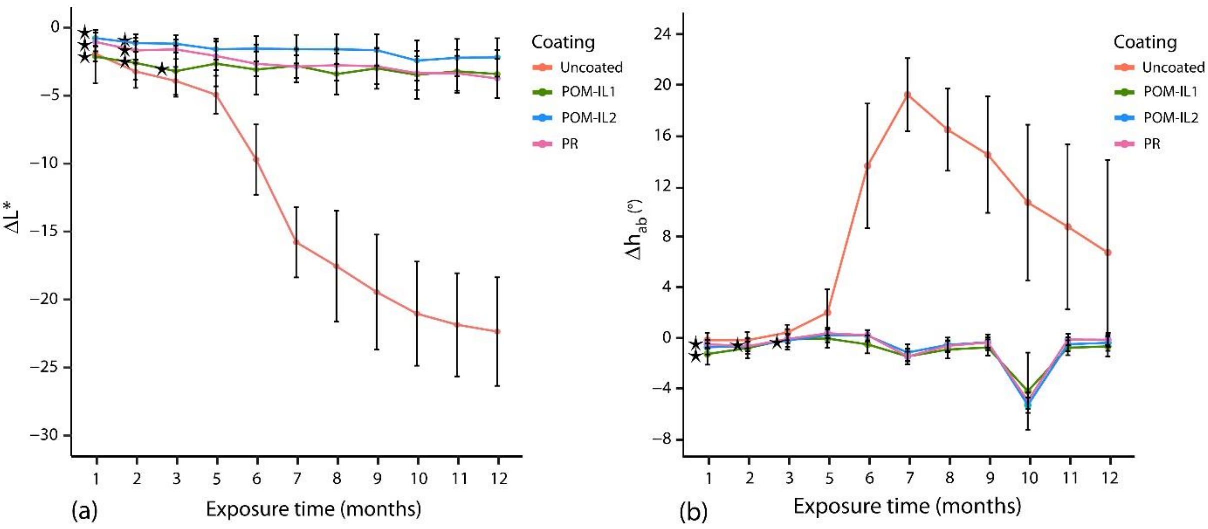

Figure S1. Graphs of ΔL* (a) and Δh_{ab} average (b) with standard deviation through 12 months of measurements of untreated (control) and treated triplicate samples (anti-colonization assay 1). For an easy reading of the graph, * corresponds to statistically NO-significant differences while no * corresponds to statistically differences between the color variation of control and each biocidal area (Fisher's test, $p < 0.05$).

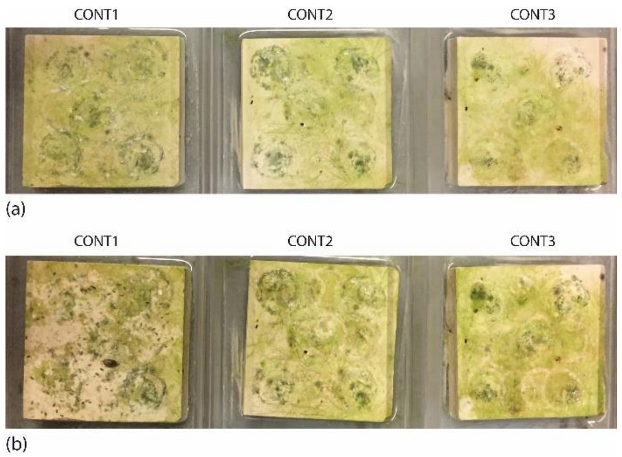

Figure S2. Uncoated control samples at month 7 (a) and month 8 with the eating of the green biofilm on CONT1 by a woodlice which could explain the fall in values from month 8.

Supplementary information

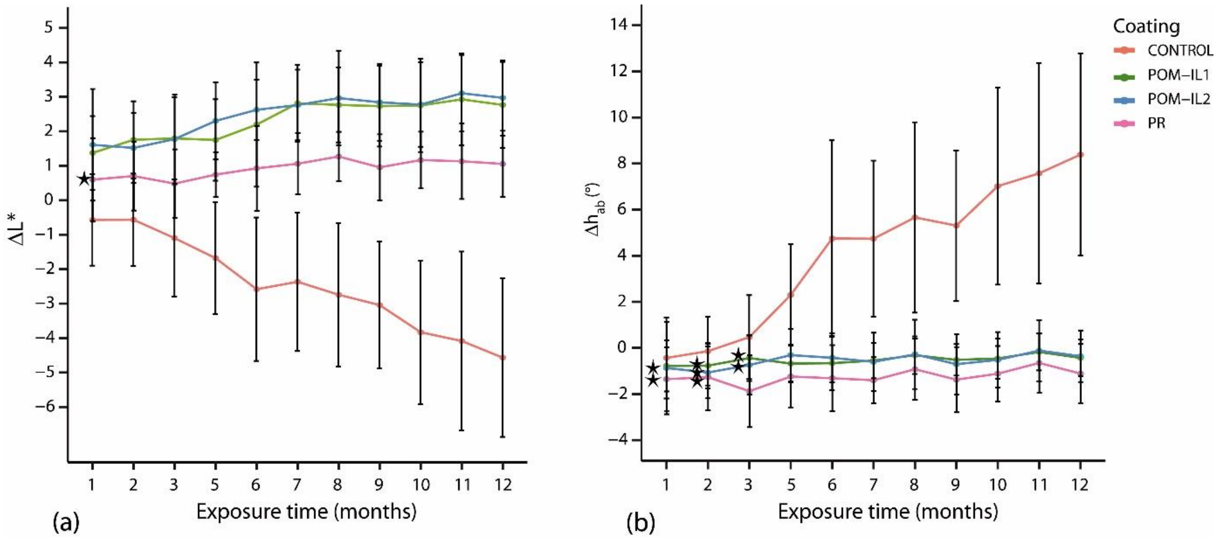

Figure S3. Graphs of ΔL* average (a) and Δh_{ab} average and standard deviation (b) through 12 months of measurements on untreated (control) and treated cleaned areas in a wall of the cellar (biofilm recolonization prevention assay 2). * in the graph corresponds to statistically NO-significant differences between the color variation of control and each biocidal area (Fisher's test, $p < 0.05$).

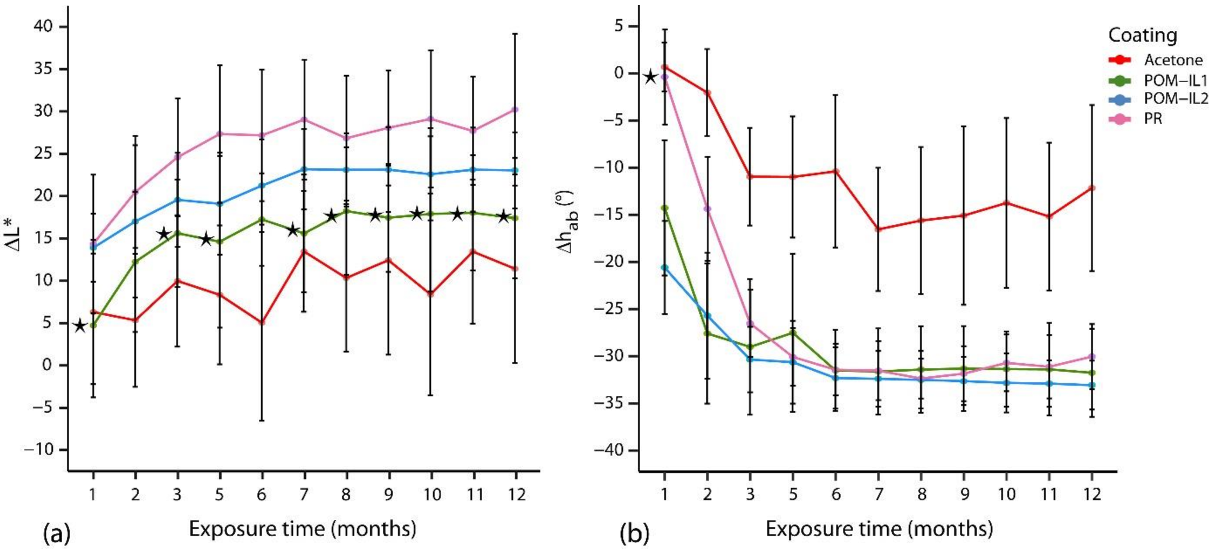

Figure S4. Graphs of ΔL* average (a) and Δh_{ab} average and standard deviation (b) through 12 months of measurements of areas treated by biocides and acetone in a wall of the cellar (biofilm removal assay 3). * in the graph corresponds to statistically NO-significant differences between the color variation of acetone and of each biocidal area (Fisher's test, $p < 0.05$).

Supplementary information

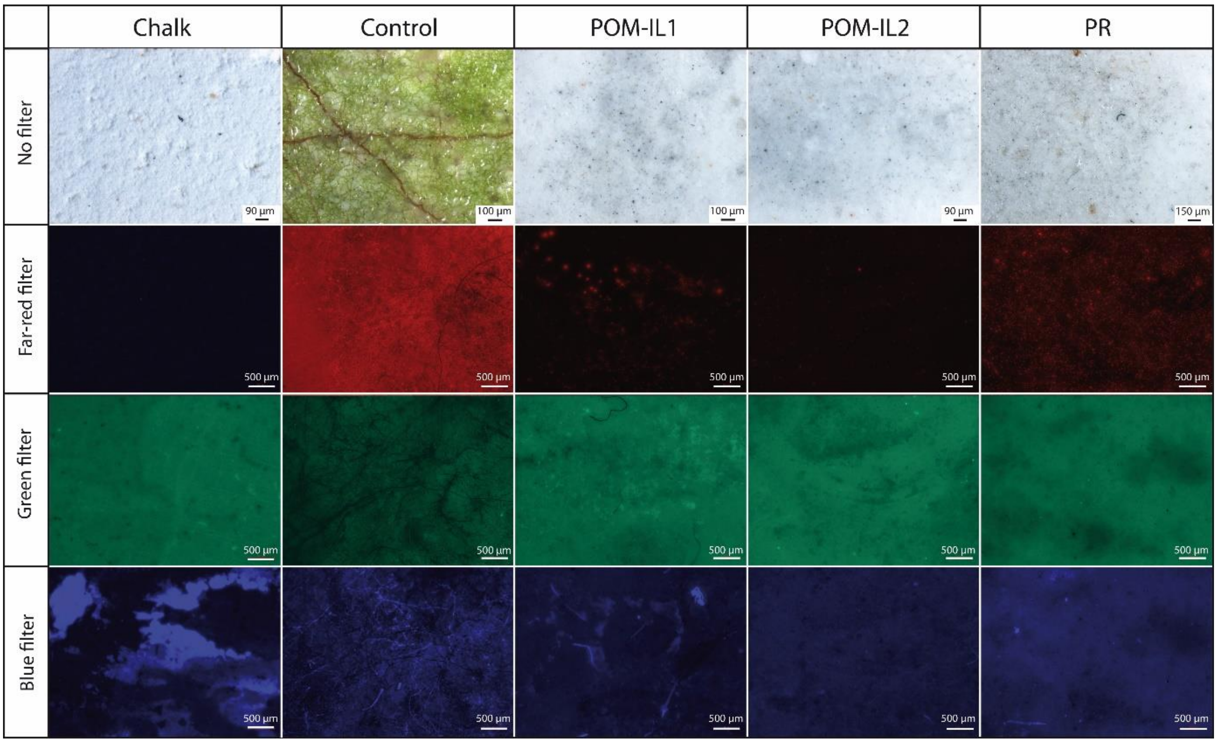

Figure S5. Confocal fluorescence macroscope images of freshly-cut chalk (Chalk) and one-year exposed chalk: uncoated (Control) or POM-IL1, POM-IL2 and PR coated samples.

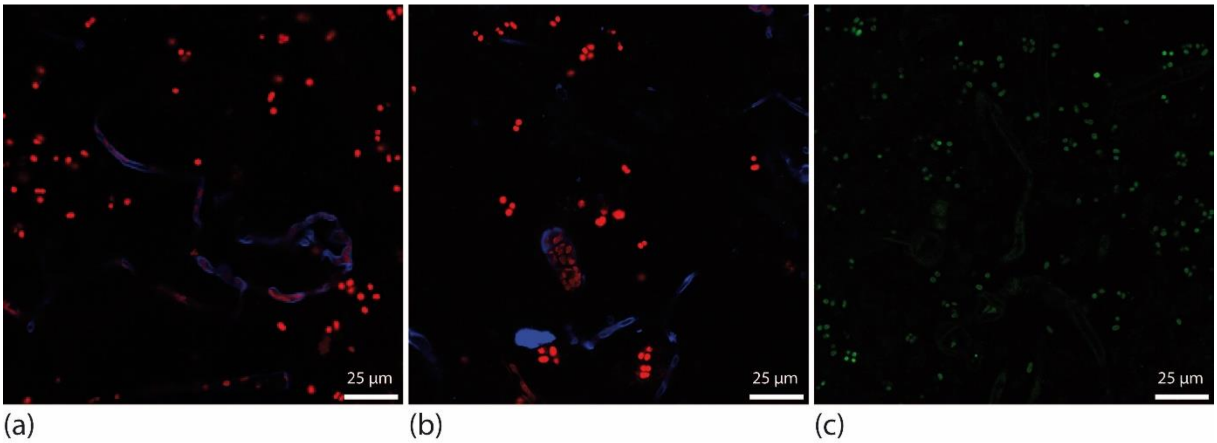

Figure S6. Confocal fluorescence microscope images of biofilm scrapped from the upper face of a control (uncoated) chalk sample after one year of exposure in a lighted room (12h light/day) of the cellar. Observations were carried out at 405 nm excitation with 415 to 460 nm emission (with a blue filter and highlighted with Calcofluor White staining), it revealed the cell membranes of moss; 638 nm excitation (with a red filter) with 648 to 678 nm emission revealed chloroplasts inside cell membranes of moss and in algae, 488 nm (with green filter) with 498 to 650 nm emission revealed cyanobacteria.

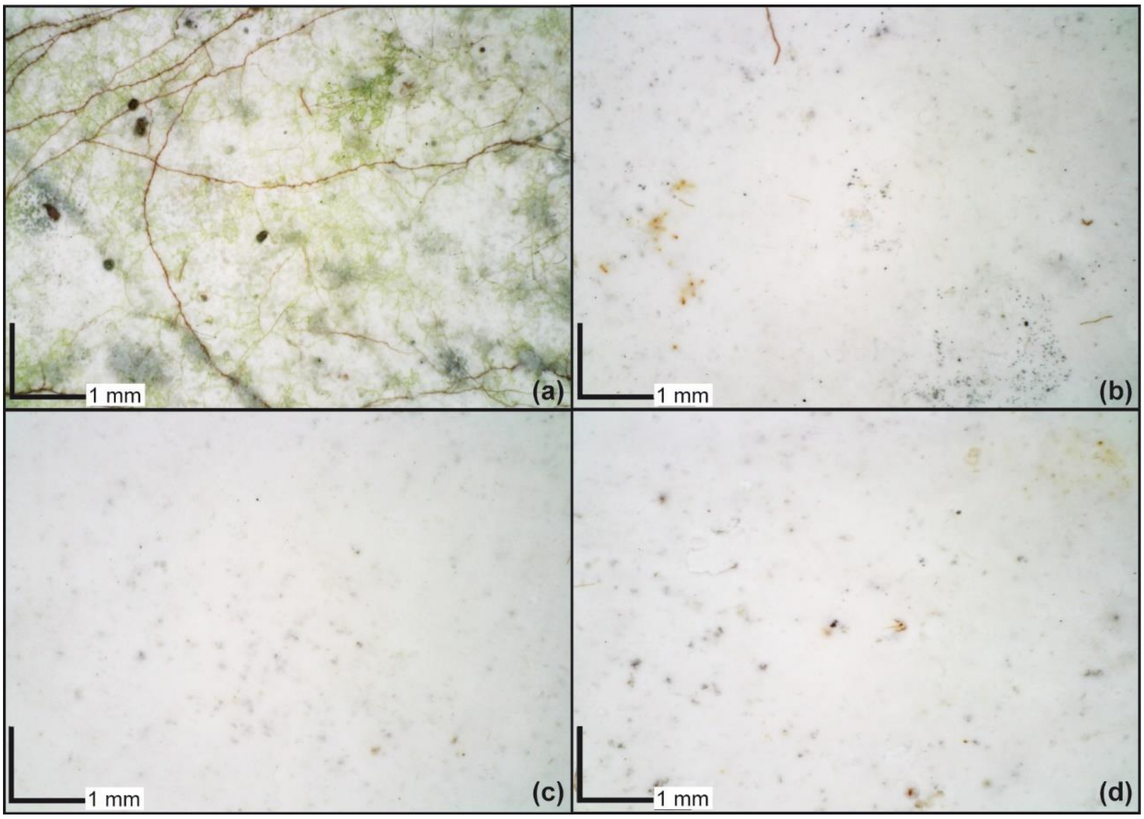

Figure S7. Microscopic observations of areas of biofilm recolonization prevention assay 2 after one year of exposure. Photos in natural light of Control area (a), PR (b), POM-IL1 (c), POM-IL2 (d).

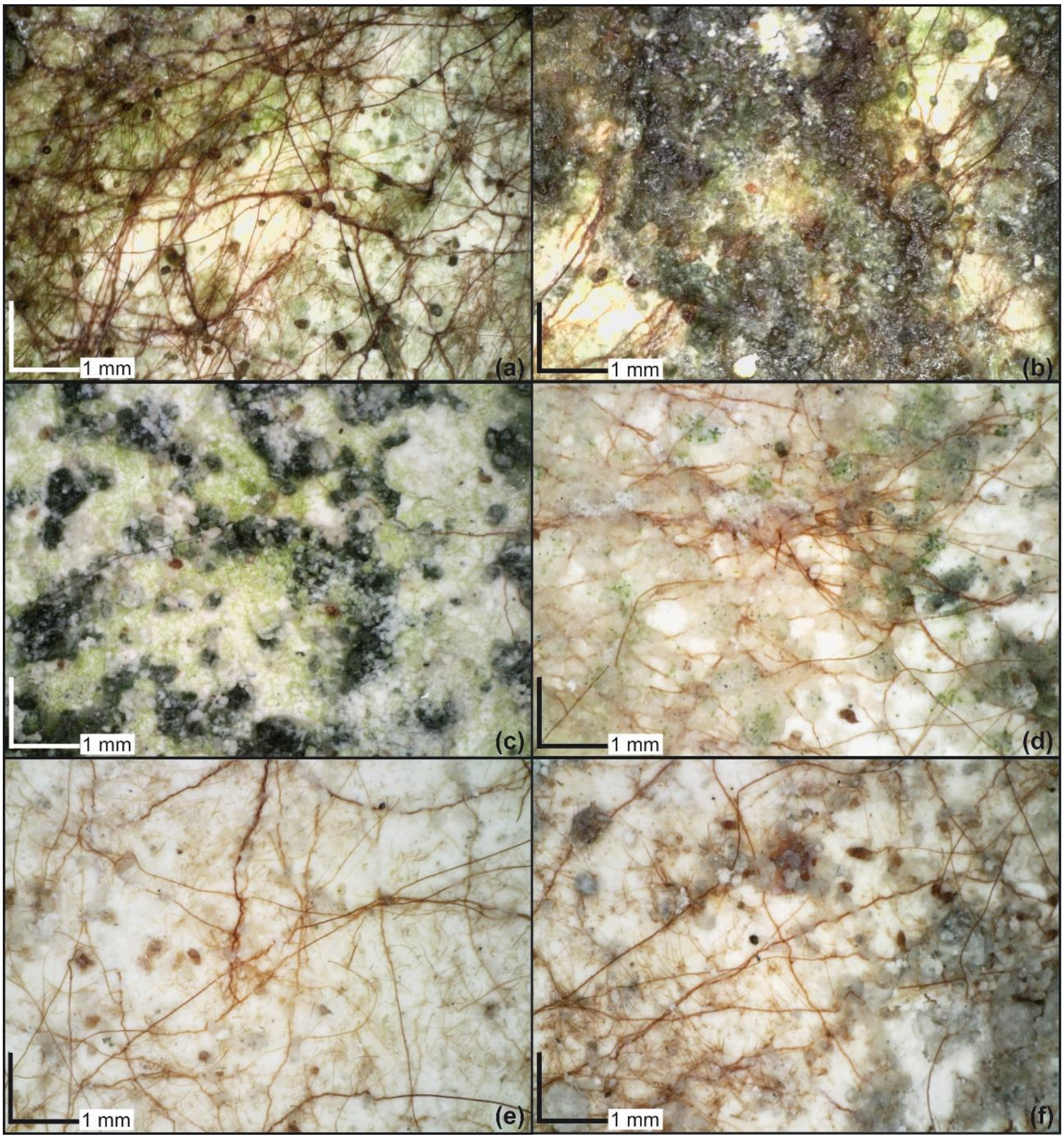

Figure S8. Macroscopic observations of areas of biofilm removal assay 3 after one year of exposure. Photos in natural light of existing biofilm (a) (b), acetone (c), PR (d), POM-IL1 (e), POM-IL2 (f).

Supplementary information

Table. S2. qPCR data with 16S rRNA, ITS1 and *rbc*L gene (cp/m^2). In Student's test carried out with 16SrRNA data, stars correspond to statistically significant differences between SC and treated areas (SR and SO1) in assay 1, between WPC and treated areas (WPR and WPO1) in assay 2 and between WB and WCO1 in assay 3. *** corresponds to $p < 0.001$, ** corresponds to $p < 0.01$, * corresponds to $p < 0.1$, no star for no statistically significant difference.

**Assay**	**Area/batch**	**16S rRNA**	**ITS1**	***rbc*L gene**
Anti-colonization	SC	$2.6.10^{10} \pm 2.8.10^{9}$	$4.6.10^{9} \pm 1.6.10^{9}$	$6.5.10^{8} \pm 9.3.10^{7}$
assay 1	SR	$1.1.10^{11} \pm 1.8.10^{10***}$		
	SO1	$1.9.10^{9} \pm 2.9.10^{8*}$		
Biofilm recolonization prevention	WPC	$9.1.10^{9} \pm 3.7.10^{8}$	$5.4.10^{8} \pm 6.0.10^{7}$	$1.5.10^{8} \pm 2.4.10^{6}$
assay 2	WPR	$8.1.10^{9} \pm 3.9.10^{9}$	$1.5.10^{7} \pm 9.2.10^{5}$	
	WPO1	$7.5.10^{9} \pm 8.7.10^{7*}$		
Biofilm removal	WB	$2.3.10^{11} \pm 2.3.10^{10}$	$3.3.10^{10}$	$9.2.10^{9} \pm 2.7.10^{8}$
assay 3	WCO1	$5.9.10^{10} \pm 7.1.10^{8***}$		

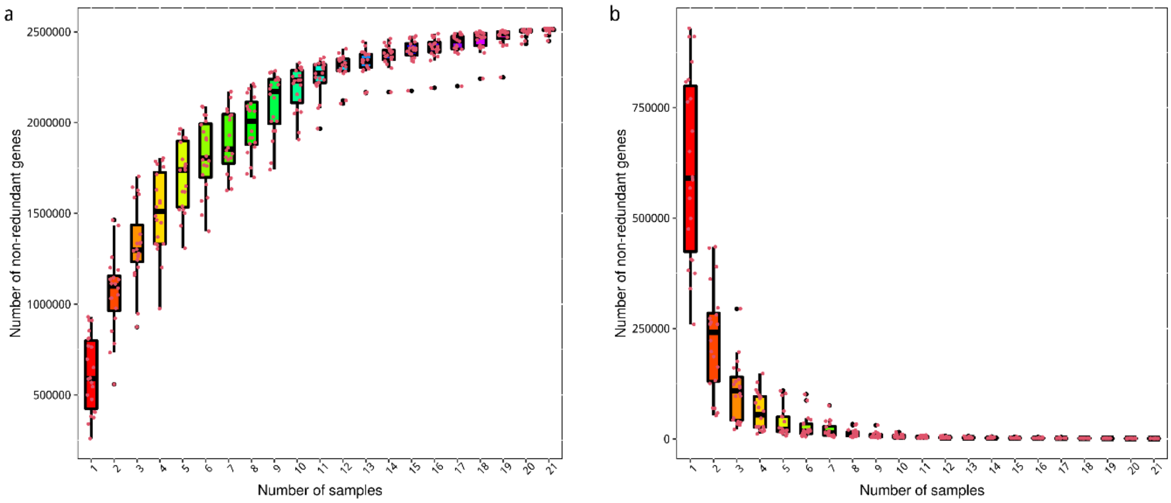

Figure S9. Rarefaction curves based on the gene abundance table, plotted with the number of core-genes and pan-genes separately by randomly drawing sampling. (a) Pan genome rarefaction curve. (b) Core genome rarefaction curve. X-axis means the number of samples that sampled. Y-axis means the gene number.

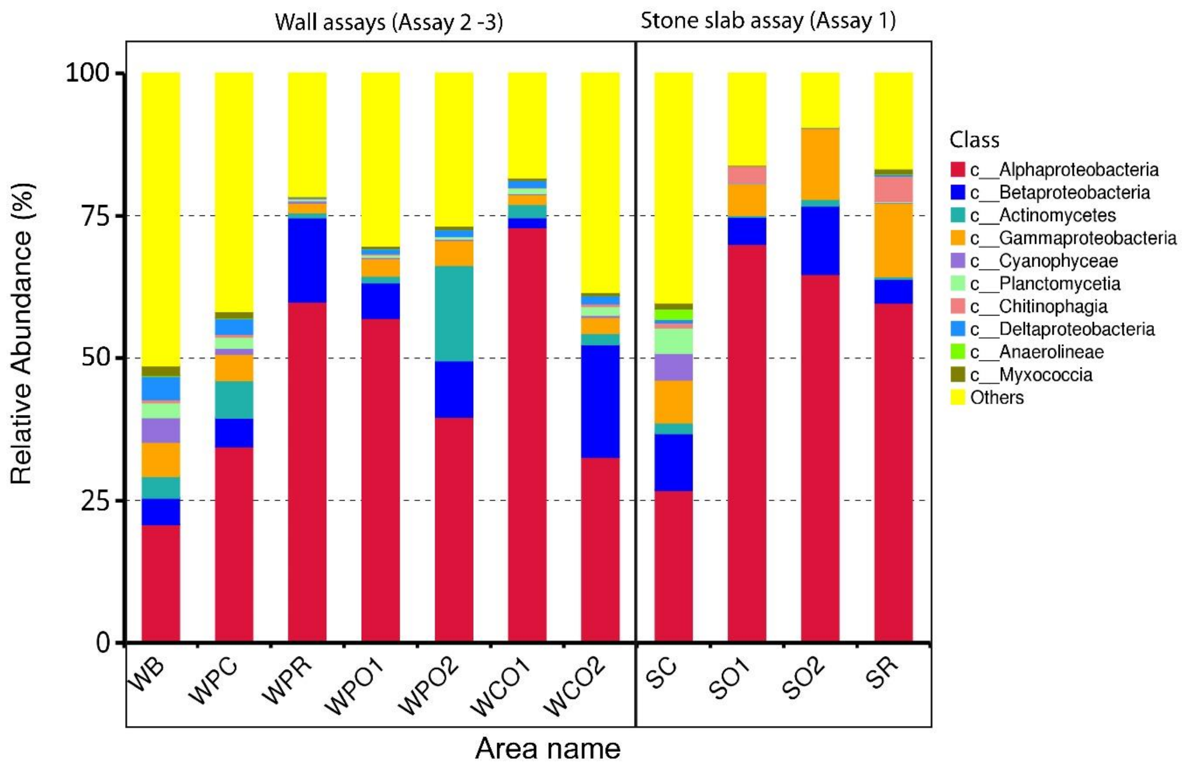

Figure S10. Relative taxonomy abundance of areas and slabs in taxonomic class level.

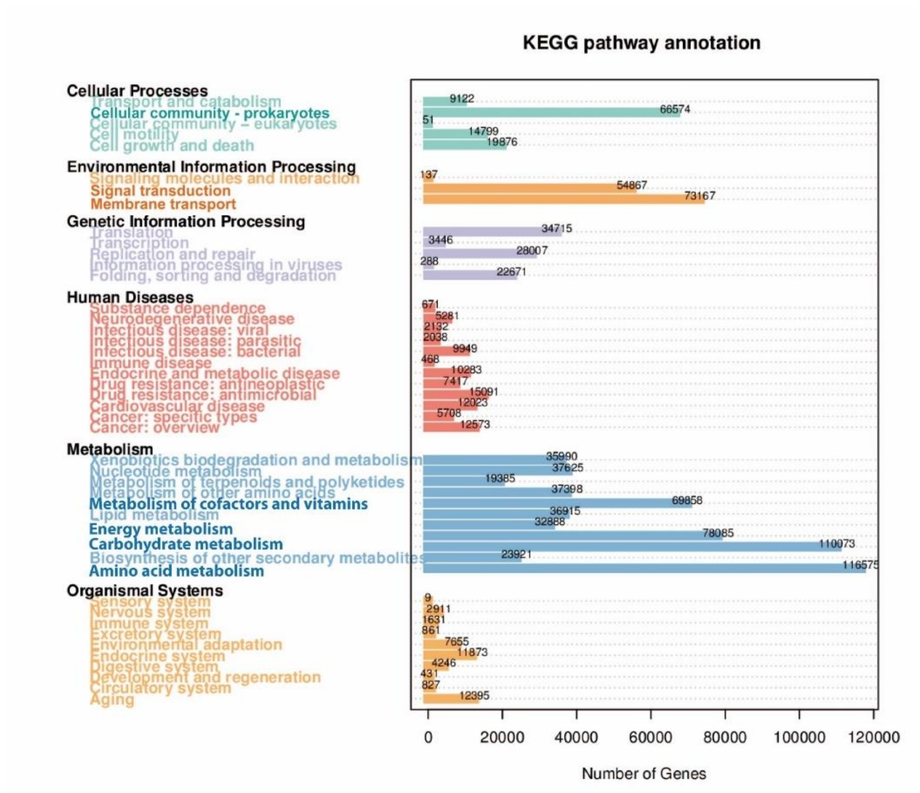

Figure S11. KEGG pathway. Summarized chart for the gene number annotated by KEGG database (Level 1).

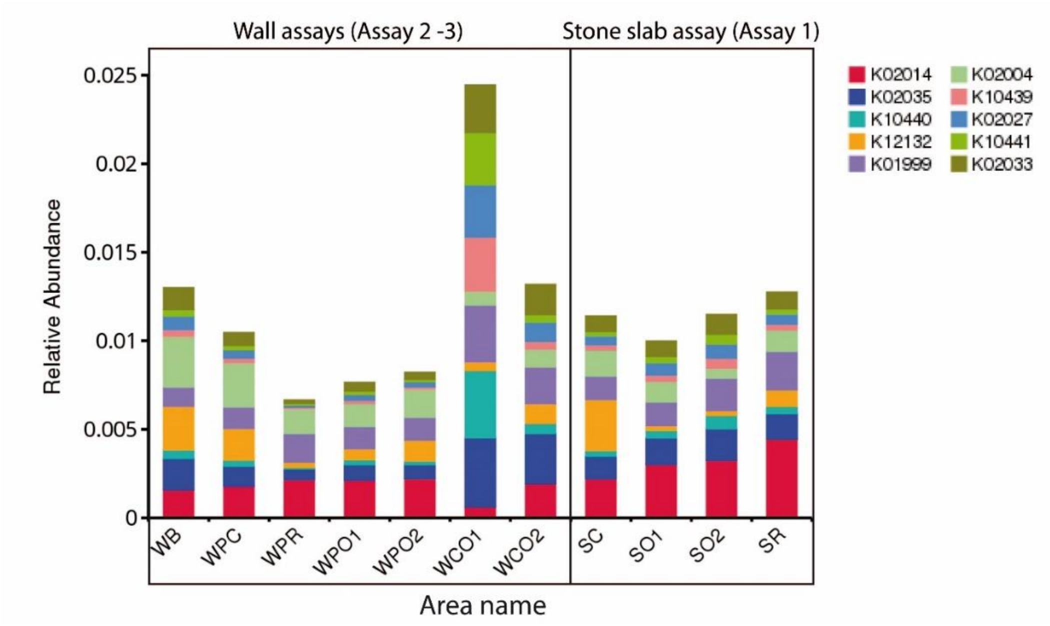

Figure S12. Relative abundance of KEGG for each area with KO database. K02014: iron complex outer membrane receptor protein ; K02035: peptide/nickel transport system substrate-binding protein ; K10440: ribose transport system permease protein; K12132: eukaryotic-like serine/threonine-protein kinase; K01999: branched-chain amino acid transport system substrate-binding protein; K02004: putative ABC transport system permease protein; K10439: ribose transport system substrate-binding protein; K02027: multiple sugar transport system substrate-binding protein; K10441: ribose transport system ATP-binding protein; K02033: peptide/nickel transport system permease protein.

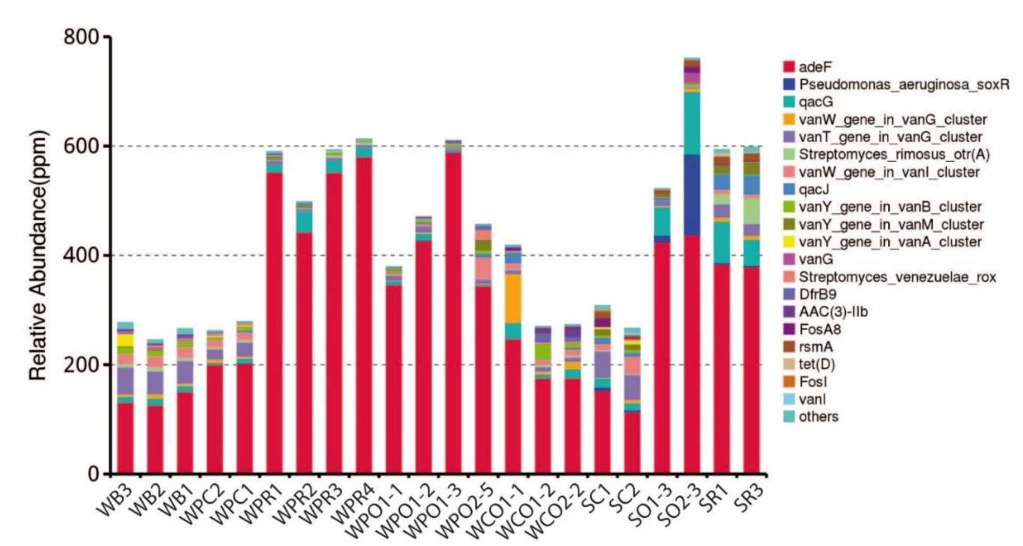

Figure S13. Relative abundance of antibiotic resistance gene. For the legend, the resistance gene with two or more resistance types are shown using abbreviations. The relative abundance in Unit ppm is the result of magnifying 10e6 times of the original abundance data.

Supplementary information

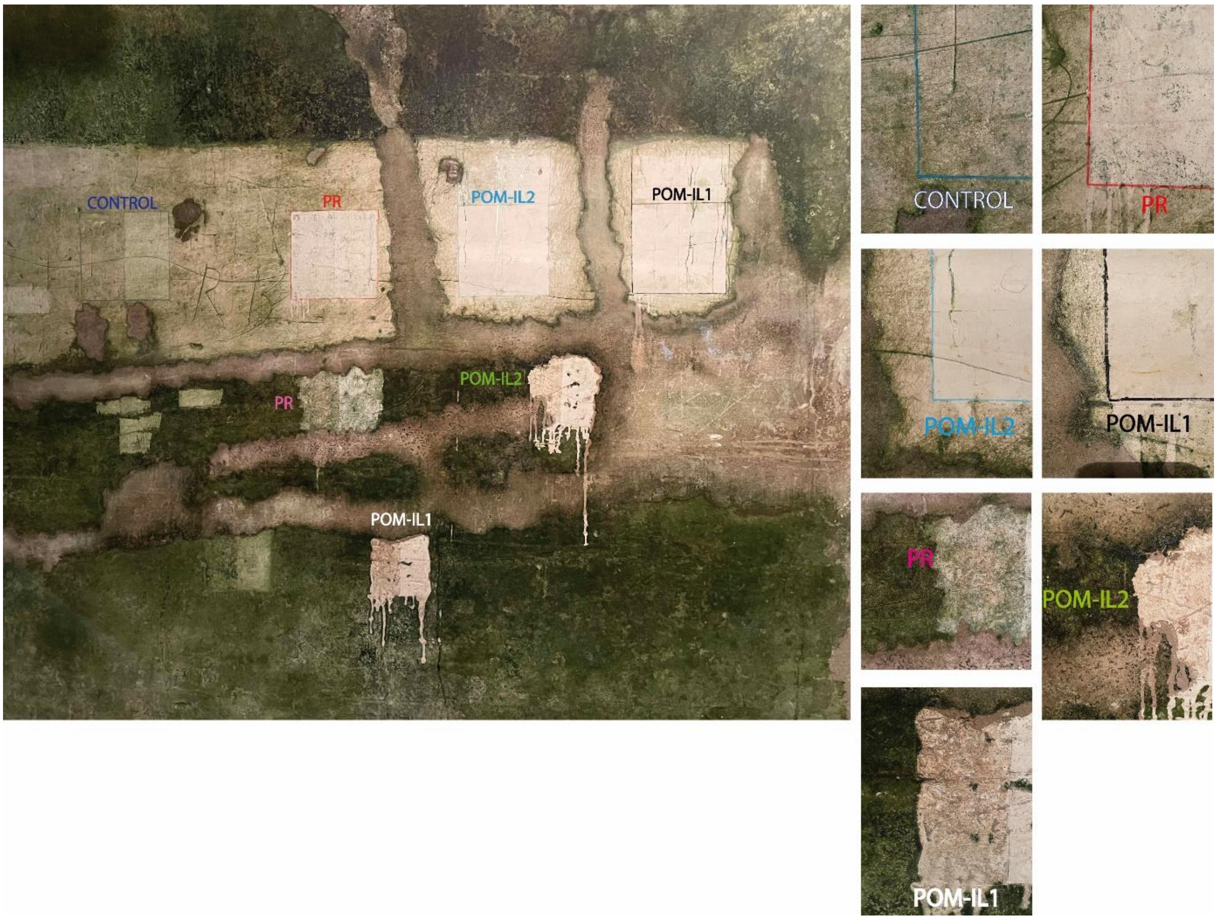

Figure S14. Photo of the wall 2.5 years after the application of products. The right side of the areas is clearer because of the scratching after one year of exposure to the light, the left side has not been scratched and corresponded to 2.5 years of exposure.
